# Immobilization of Chromium by Iron Oxides in Nickel–Cobalt Laterite Mine Tailings

**DOI:** 10.1021/acs.est.4c05383

**Published:** 2025-03-14

**Authors:** Ruth Esther Delina, Jeffrey Paulo H. Perez, Vladimir V. Roddatis, Jessica A. Stammeier, Damien Prieur, Andreas C. Scheinost, Mark M. Tan, Jhonard John L. Garcia, Carlo A. Arcilla, Liane G. Benning

**Affiliations:** † GFZ Helmholtz Centre for Geosciences, Telegrafenberg, Potsdam 14473, Germany; ‡ Department of Earth Sciences, Freie Universität Berlin, Berlin 12249, Germany; § The Rossendorf Beamline at ESRF, The European Synchrotron, CS 40220, Grenoble 38043, Cedex 9, France; ∥ Institute of Resource Ecology, Helmholtz-Zentrum Dresden-Rossendorf, Bautzner Landstraβe 400, Dresden 01328, Germany; ⊥ National Institute of Geological Sciences, University of the Philippines, Quezon City, Diliman 1101, Philippines; # Department of Science and Technology, Philippine Nuclear Research Institute, Quezon City, Diliman 1101, Philippines

**Keywords:** mine waste, high pressure acid leaching, coprecipitation, hematite, chromite, sequential extraction, X-ray absorption spectroscopy

## Abstract

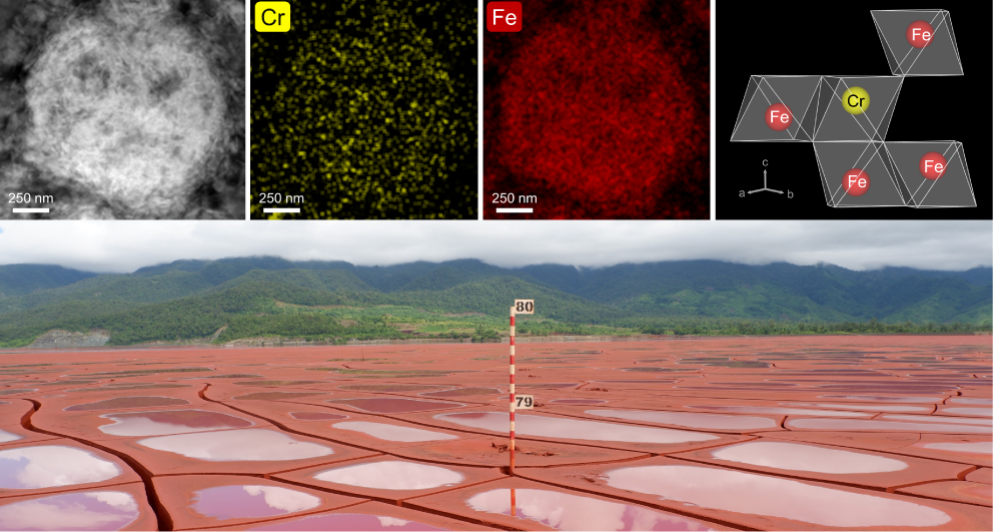

Mine tailings generated from hydrometallurgical processing of nickel–cobalt laterite deposits contain high levels of chromium (Cr), with the hexavalent species being a toxic pollutant and carcinogen. However, the partitioning, speciation, and local bonding environment of Cr in the mine tailings remain largely unknown, hindering our ability to predict its toxicity and long-term behavior. Coupling detailed mineralogical, spectroscopic, and geochemical characterization with sequential extraction of tailings from active and rehabilitated dams, we show that Cr is present in its least toxic form, Cr(III), and largely immobilized by recalcitrant minerals. This immobilization also regulates dissolved Cr concentrations in the interacting waters to levels up to five times lower than the global regulatory limit (50 μg L^–1^). Solid-phase Cr concentrations were ≤1.5 wt % with 39–61% of Cr incorporated into hematite, and to a lesser extent, alunite, both of which formed early in the hydrometallurgical extraction process of mined laterite ores. The remaining Cr was present as recalcitrant chromite residues from the primary source laterites. We highlight that, although hydrometallurgical extractions liberate Cr from laterite ores during processing, they also provide ideal chemical pathways for the formation of highly stable, crystalline hematite that successfully sequesters Cr, while restricting its environmental mobility.

## Abstract 2

We show that in nickel−cobalt laterite mine tailings, the structural incorporation of chromium in secondary iron (oxyhydr)oxides immobilizes the majority of the chromium that is released during ore processing.

## Introduction

Global transition to cleaner energy sources has raised the demand for critical metals,^1^ exponentially also increasing the amount of waste generated from their extraction.^2^ Over half of the world’s nickel (Ni) resources are hosted in laterite deposits,^3^ and the extraction of Ni as well as cobalt (Co) from these ore deposits results in millions of tons of tailings posing various environmental issues. Given the occurrence of potentially toxic heavy metals (e.g., chromium) in the source laterite ores,^4−6^ the treatment, storage, and disposal of associated mine wastes remain a significant challenge for the Ni industry.^7−9^

Chromium (Cr) is a common constituent of Ni–Co laterite deposits, occurring as Cr(III) incorporated in minerals such as primary Cr-spinels (e.g., chromite) and laterite weathering phases like Fe (oxyhydr)oxides, predominantly goethite but also hematite and poorly crystalline phases.^10−13^ Chromium may also be present as Cr(VI) oxyanions adsorbed onto these weathering mineral surfaces.^14,15^ The presence and chemical nature of Cr in Ni–Co laterite ores are actively studied,^4,6,16^ but its fate during ore processing and later in the mine wastes or tailings remains largely unknown. Understanding its fate is critical because Cr in mine tailings can adversely affect surrounding environments and water bodies, depending on its chemical speciation. Although in its Cr(III) state, it is a vital micronutrient,^17^ Cr(VI) is carcinogenic.^18−20^ Hexavalent Cr is more mobile than Cr(III), and its mobilization in Ni–Co laterite mining areas and potential transfer to and toxicity in ground- and drinking waters worldwide is of huge concern. In the Philippines and New Caledonia, for example, where laterite mines are exposed to tropical climates, Cr(VI) contents in surface waters (up to 143 and 1620 μg L^–1^, respectively)^4,6^ occasionally exceeded international drinking water standards (i.e., World Health Organization (WHO) limit of 50 μg L^–1^) by several magnitudes.^4−6^ However, the role of mine tailings in the remobilization of Cr in laterite areas remains unclear. With the consistent increase in the global Ni and Co production from laterite deposits,^21^ understanding the fate of Cr in mine tailings is necessary for the environmental management and sustainable development of lateritic regions.

In most worldwide Ni–Co laterite processing plants (e.g., in New Caledonia, Australia, Philippines), high-pressure acid leaching (HPAL) is the primary hydrometallurgical method used to concentrate Ni and Co from iron (Fe) (oxyhydr)oxide-rich limonite or oxide-type laterite deposits (Figure S1).^22^ Nickel and Co mainly hosted in Fe (oxyhydr)oxides, such as goethite, are leached using sulfuric acid (H_2_SO_4_) under high temperature (230–270 °C) and pressure (3.3–5.5 MPa) conditions. Nickel and Co, simplified in the form of oxides, dissolve and remain in solution according to eqs 1 and 2, respectively.
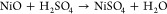
1
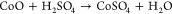
2

Meanwhile, Fe follows a dissolution–precipitation path resulting in secondary Fe (oxyhydr)oxides (eqs 3 and 4) and sulfates.^22,23^
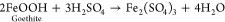
3
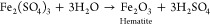
4

The resulting acid leach residue is neutralized by limestone (CaCO_3_) or lime (CaO) addition,^22,24,25^ forming calcium (Ca) sulfates as byproduct.^7^ This neutralized slurry is typically discharged to a tailings dam or storage facility.^7,25^Figure S1b and Table S1 provide an overview of the HPAL process and selected literature data on HPAL residues from processing plants and laboratory-scale experiments, respectively. HPAL tests on different laterite ores showed that up to 25% of the total Cr content could be leached,^23,26,27^ and under oxidizing HPAL conditions, this dissolved Cr has been predicted to exist as Cr(VI), either as CrO_4_^2–^ or Cr_2_O_7_^2–^.^22,26,28^ However, there is no direct evidence yet for the specific speciation and mobility of Cr in the acid leach residues that end up downstream as tailings, raising critical questions about its environmental behavior, toxicity, and risk potential in Ni–Co laterite mining regions. More importantly, current research has not quantitatively evaluated the potential of Cr to leach from tailings into waterways, although such information is essential for assessing environmental impacts and informing management strategies to mitigate possible Cr pollution.

To address this knowledge gap, we have carried out detailed mineralogical, spectroscopic, and geochemical characterization of tailings from active and rehabilitated HPAL tailings dams in a mining district in the Philippines. We coupled these findings with targeted sequential extraction in order to determine the host minerals, the Cr speciation, as well as the fate and mobility of Cr in these environments. Our findings enable us to predict the long-term behavior of Cr in Ni–Co laterite mine tailings, and to assess its implications for tailings management and disposal.

## Materials and Methods

### Sample Collection and Field-Based Measurements

HPAL tailings (solids and water samples) were collected in May 2022 from two sites in Palawan, Philippines: (1) an active tailings dam and (2) an adjacent rehabilitated tailings dam that was decommissioned for over 10 years (Figure S2). Sampling and *in situ* measurements were conducted at safe and accessible locations free from infrastructure and allochthonous material that could potentially influence or alter the tailings. The solid samples can be divided into (i) wet or waterlogged tailings (AW – active wet) and (ii) dry tailings (AD – active dry) from the active tailings dam, and (iii) rehabilitated (RH) tailings from the decommissioned tailings dam (Figure S2). Samples were collected by digging small pits and removing ∼10 cm of surface cover. The field moist samples were stored in polyethylene bags, then freeze-dried and ground upon arrival in the laboratory. Water samples were obtained from the waterlogged areas of the active tailings dam and from its outflow pond. We measured temperature, pH, redox potential (ORP), electrical conductivity (EC), and dissolved oxygen (DO) *in situ* using freshly calibrated HACH HQd and Hanna HI portable multiparameter meters. Duplicate water samples were collected in low-density polypropylene bottles that were filled to the brim, capped tightly and stored at ∼4 °C during transport to the laboratory, where the samples were then stored in a refrigerator prior to analyses. A batch of water samples was filtered through Nalgene Rapid-Flow filter units using 0.2-μm PES membranes. The filtrate was split into unacidified subsamples for Cr(VI) and anion measurements and acidified (0.3 M HNO_3_) subsamples for cation analysis. The second batch was used for alkalinity measurements.

### Aqueous Phase Analyses

Dissolved Cr(VI) concentrations (LoD = 10 μg L^–1^) were determined within 24 h of sampling using a Shimadzu UV 1800 UV–vis spectrophotometer and following the USEPA 7196A^29^ method. Total Cr (LoD = 14 μg L^–1^) and major element concentrations in all water samples were measured by inductively coupled optical emission spectrometry (ICP-OES Varian 720-ES).^10^ Dissolved anion concentrations were quantified by ion chromatography (Metrohm AG – 883 Basic IC plus) and alkalinity was analyzed by titration following the USEPA 310.1^30^ method. Based on the water chemistry data, mineral saturation indices were calculated using the Geochemist’s Workbench.^31^

### Mineralogical and Geochemical Characterization

The bulk chemical composition of the solids was quantified by ICP-OES (Agilent 5110) analysis following total digestion of solid aliquots prepared by Na_2_O_2_ fusion, and data is reported as oxide (wt %) following standard methods.^32^ The analytical uncertainty of this approach was determined using OREAS certified reference materials (CRMs) 182, 185 and 190, all nickel laterite ores. These CRMs were within 10% of the certified values. The mineralogical composition of all samples was quantified from patterns recorded with a STOE STADI P X-ray diffractometer (XRD, Ag Kα radiation; λ = 0.5594 Å) equipped with a curved Ge (111) monochromator and two DECTRIS MYTHEN2 R detectors. XRD patterns were collected over a *Q*-range of 0 to 13.42 Å^–1^, and data was processed by Rietveld refinement using the GSAS-II software.^33^ Particle size distributions were determined using a Horiba/Retsch LA950 laser diffraction particle size analyzer. Prior to analysis, the samples were placed in an ultrasonic bath for 15 min to mechanically disaggregate particles. The particle morphologies and elemental compositions of solids were examined using a FEI Quanta 3D FEG scanning electron microscope (SEM) coupled with an EDAX energy dispersive spectroscopy (EDS) system. SEM images of the carbon-coated (20 nm) samples were acquired at high vacuum mode, and at 20 keV and 4 nA using an Everhart-Thornley secondary electron detector (ETD) and a backscattered electron detector (BSED). For selected samples, thin foils were prepared by Ar^+^ ion milling using a Gatan PIPS II 695 setup and a focused ion beam milling (FIB) system using a FEI Helios G4 UC Dual Beam FIB-SEM instrument. On these FIB foils, high angle annular dark-field scanning transmission electron microscopy (HAADF-STEM) micrographs and energy dispersive X-ray (EDX) maps were acquired using a Thermo Fisher Scientific Themis Z (3.1) Scanning Transmission Electron Microscope operated at 300 kV and equipped with a Super-X EDX system and a Gatan Continuum ER/1065 imaging filter.

### Cr Speciation and Bonding Environment

To evaluate the speciation and local bonding environment of Cr in the tailings, we employed synchrotron-based X-ray absorption spectroscopic (XAS) analyses of the solid fractions. Cr–K edge XAS was conducted at the Rossendorf Beamline (ROBL-II) BM20^34^ of the European Synchrotron Radiation Facility (ESRF, Grenoble, France). XAS spectra were collected in fluorescence mode using an energy-dispersive 18-element Ge detector out to a wavevector value of 11.5 Å^–1^. Energy calibration was done by measuring a Cr foil and setting the position of the first maximum of the first derivative to 5989 eV. The collected spectra were compared with Cr-bearing reference mineral standards (e.g., Cr(III)- and Cr(VI)-ferrihydrites, Cr-hematite, chromite; Figure S3). Pressed pellets (13 mm) of the samples and standards diluted in cellulose were analyzed in a closed-cycle He cryostat (∼15 K) to minimize beam damage that can potentially change the speciation of Cr and to eliminate the thermal component of the Debye–Waller term. Dilution factors for the pellet mixtures were calculated using XAFSmass.^35^ For each sample, two to four scans were collected to obtain a good signal-to-noise ratio. Spectra were aligned, averaged, and background-subtracted using the ATHENA^36^ and SIXpack^37^ software. Details of the synthesis of Cr-hematite and the preparation of chromite can be found in Delina et al.,^10^ while the synthesis of the Cr-ferrihydrite standards and further information on data analysis of the XAS spectra can be found in Texts S1–S3.

### Cr Sequential Extraction

To evaluate the different chemical and mineral fractions that could influence the potential mobility and toxicity of Cr in the solid tailings, we employed a Cr sequential extraction procedure (SEP) previously developed for Fe-rich laterites, soils and sediments.^23^ To account for the presence of large proportions (up to 34%) of water-soluble minerals like gypsum in the solids, we slightly modified the SEP and included an additional water-soluble extraction step. Thus, our 7-step SEP discriminated between S1: water-soluble, S2: exchangeable, S3: adsorbed, S4: carbonate, S5: poorly crystalline Fe (oxyhydr)oxide S6: crystalline Fe (oxyhydr)oxide, and S7: organic matter associated Cr fractions (Table S2) with the residual fraction associated with recalcitrant chromite.

**Figure 1:**
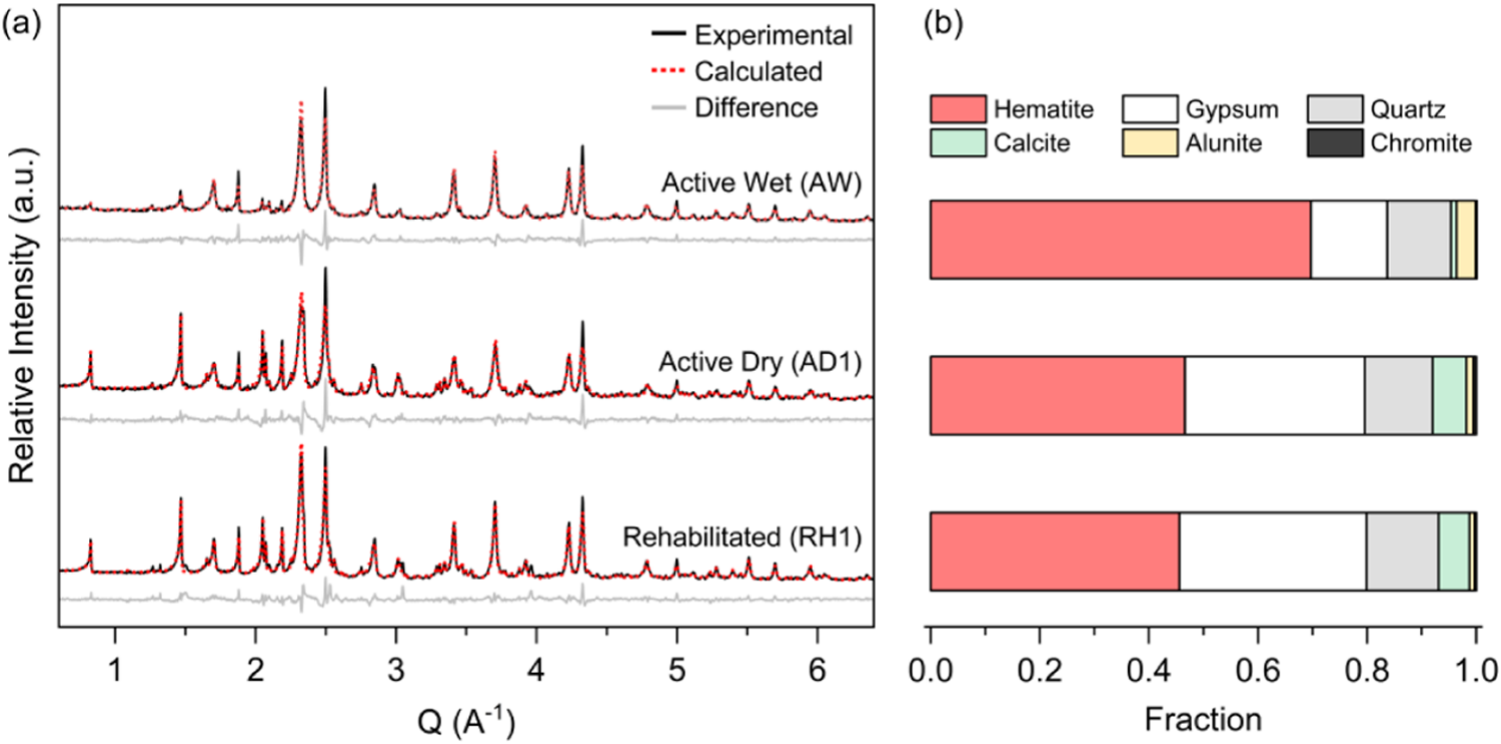
(a) XRD patterns and Rietveld refinements of representative samples from the active wet (AW), active dry (AD1), and rehabilitated (RH1) tailings; experimental data (solid black line); fitted data (dashed red line); and difference profiles (solid gray line). (b) Proportions of mineral phases of AW, AD1, and R1 based on Rietveld refinements. The XRD patterns of all samples can be found in Figure S4.

## Results

### Solid-Phase Cr Geochemistry and Mineralogy

**Figure 2:**
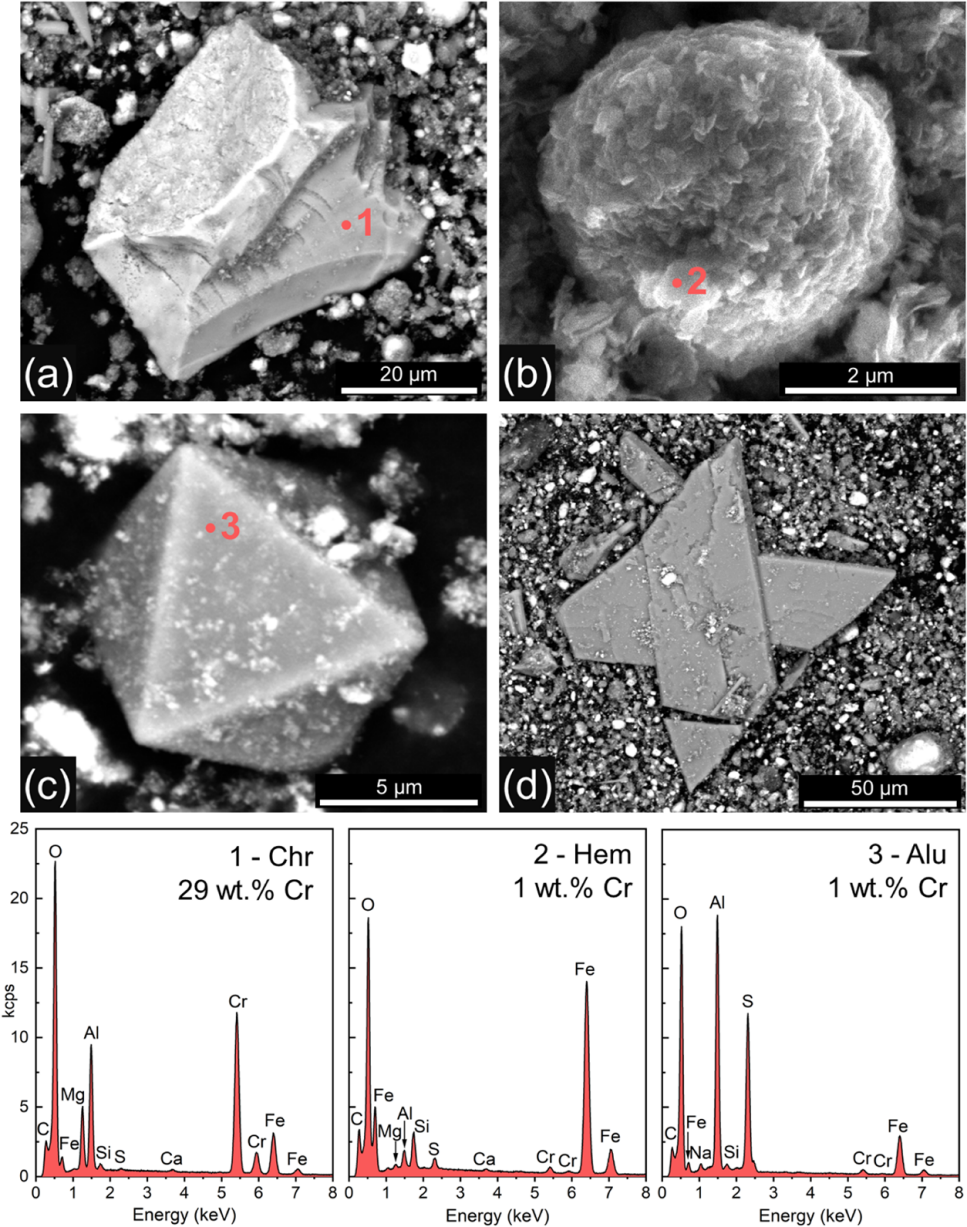
SEM images of selected minerals found in the tailings: (a) a relic chromite (Chr) grain from sample AD3, with a pitted and fractured appearance indicative of past extractive processes (e.g., milling), (b) an aggregate of nanosized platy hematite (Hem), (c) a pseudo-octahedral crystal of alunite (Alu) from sample AW, and (d) a relatively large twinned gypsum crystal found in sample AD3. Below the images are the corresponding EDS spectra and Cr concentrations of the numbered points on the Cr-bearing minerals (a–c).

Solid-phase Cr concentrations in the studied tailings ranged from 1.0 to 1.5 wt % (Table S3), comparable to values (0.5–1.3 wt %) reported for other HPAL residues worldwide (e.g., Indonesia,^38^ New Caledonia,^39^ Australia,^40^ Turkey^27^) (see also Table S1). The tailings contained high concentrations of Fe_2_O_3_ (41–53 wt %) as well as SiO_2_ (7.9–18 wt %) and minor Al_2_O_3_ (2.7–4.0 wt %) and MgO (1.5–3.4 wt %). Significant concentrations of CaO (3.7–12 wt %) from the neutralizing agents were also detected from the tailings. These compositions and, in particular, the high Fe contents were confirmed by our XRD analysis (Figure 1) that revealed high proportions of hematite (Fe_2_O_3_; 46–70%), gypsum (CaSO_4_·2H_2_O; 14–34%), quartz (SiO_2_; 12–13%), with minor (<10%) calcite (CaCO_3_), alunite ((K,Na)(Al,Fe)_3_(SO_4_)_2_(OH)_6_), and chromite ((Fe,Mg)Cr_2_O_4_; <1%). The latter pitted and fractured chromite (Figure 2a) is clearly a relic mineral from the HPAL processing. The diverse minerals in the mine tailings result in a broad particle size distribution, marked by three primary modes (Figure S5). The predominant nanosized platy crystals of hematite typically occur as matrix and aggregates (Figure 2b) that account for the size ranges from 0.1 to 1 μm and >1 μm. Discrete pseudo-octahedral crystals of alunite (Figure 2c) with lengths ranging from 5 to 20 μm and relic chromite grains with an average size of ∼50 μm (Figure 2a) contribute to the >1–80 μm size fraction while the larger tabular and sometimes twinned crystals of gypsum (Figure 2c) explain grain sizes over 80 μm. The particle size distributions and mineral compositions indicate that, among the tailings, the waterlogged sample is marked by its higher hematite content and the smallest amount of gypsum, likely due to the undersaturation of gypsum in the reacting waters (Table S4). For all other solids, we observed only minor differences in mineralogical compositions.

Electron micrographs and spectral analyses of the tailings (Figure 2) indicated that Cr is primarily associated with chromite, hematite, and alunite crystals. Chromite was the only discrete Cr-bearing mineral found, with Cr concentrations between 23 and 45 wt % (SEM-EDS analyses; Table S5). SEM-EDS point analysis of hematite grains yielded Cr concentrations ranging from 0.5 to 1.3 wt %, close to values measured from alunite crystals (0.3–1.1 wt %) (Table S5).

**Figure 3:**
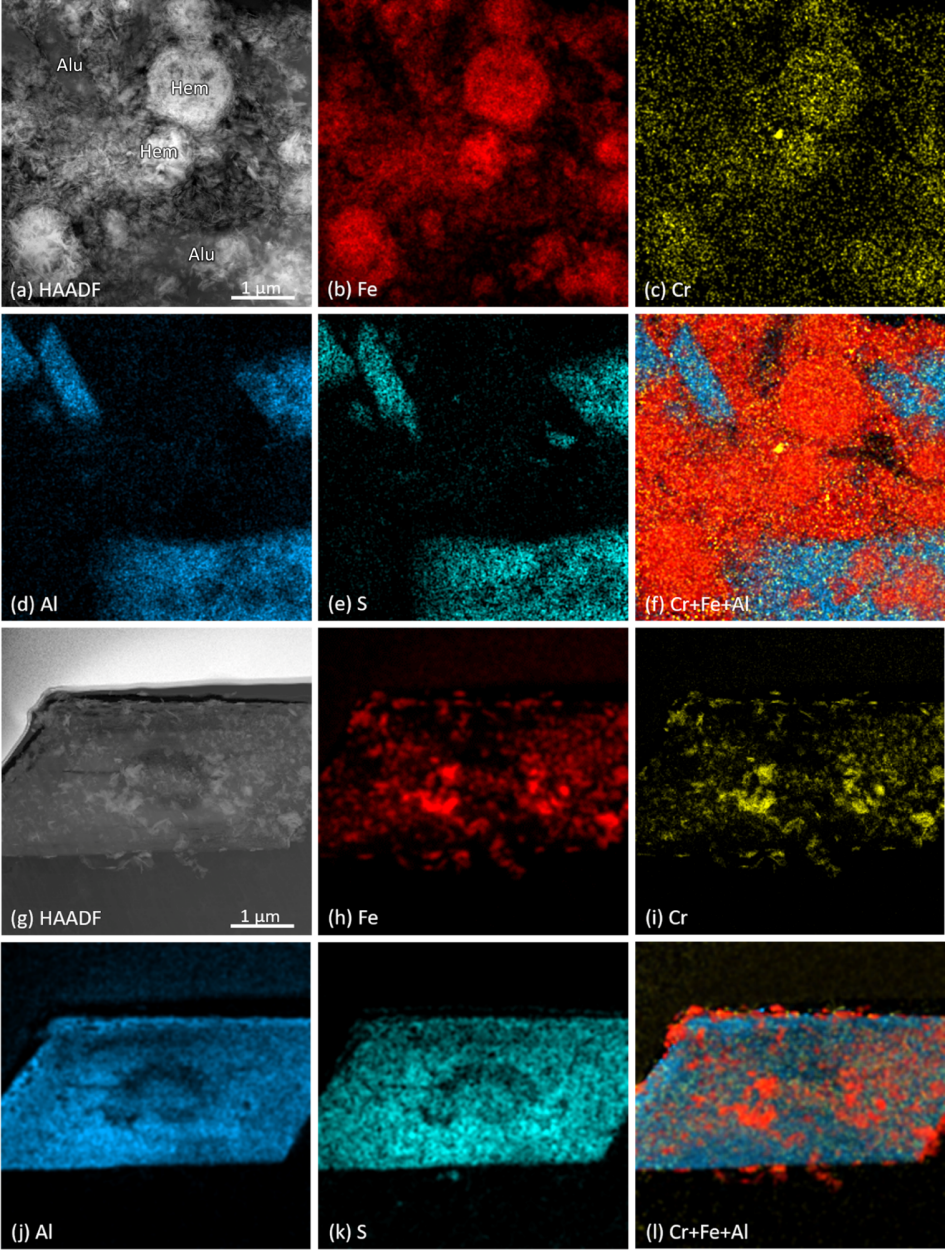
HAADF-STEM images of (a) hematite (Hem) aggregates and matrix with alunite (Alu), and (g) a FIB section of an alunite crystal. The corresponding EDX elemental maps are shown for: (b,h) Fe (red); (c,i) Cr (yellow); (d,j) Al (blue); (e,k) S (cyan); and (f,l) combined Cr, Fe, and Al.

Elemental distribution maps acquired by STEM-EDX of the tailings (Figure 3a–e) confirmed that Cr (yellow), was closely associated with the hematite aggregates and the Fe-rich matrix (red). There was also an overlap between Cr and the Al- and S-rich lath-shaped sections of alunite (blue and cyan, respectively), supporting the SEM-EDS observations. However, the combined EDX elemental maps of Cr, Fe and Al (Figure 3f) clearly indicate higher Cr intensities associated with hematite (i.e., Cr + Fe = orange), compared to alunite (i.e., blue only). Nevertheless, it remained unclear if alunite or hematite is the prime Cr host. Thus, we analyzed FIB sections through an alunite crystal (Figure 3g) and could confirm in the EDX maps (Figure 3h–l) that Cr displayed a stronger affinity to associated Fe oxides (orange hotspots in Figure 3l) compared to alunite.

### Chromium XAS Analysis

**Figure 4:**
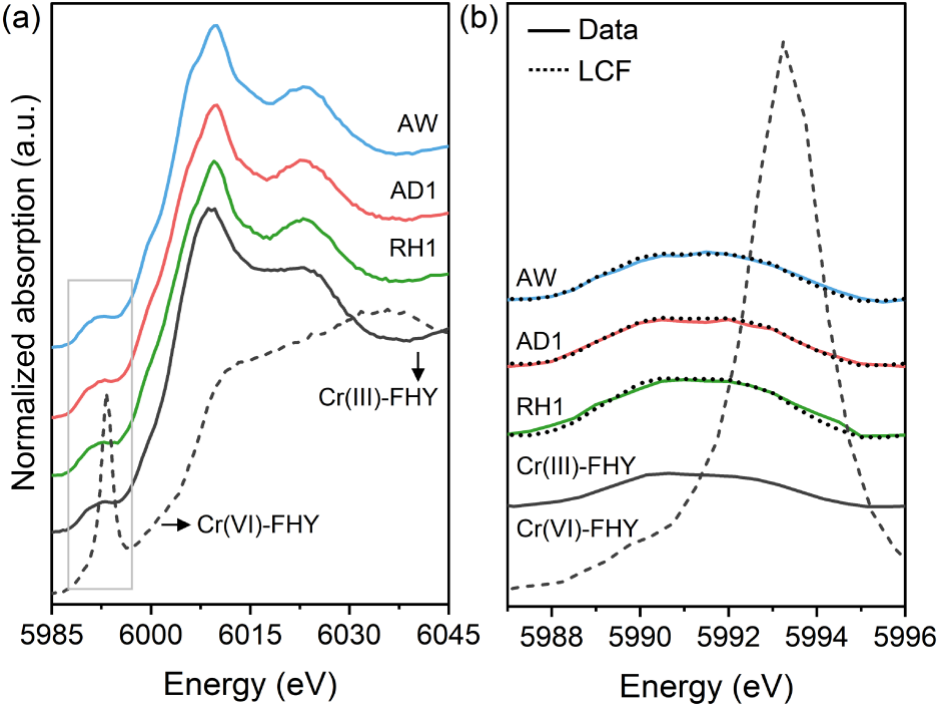
(a) Normalized Cr K-edge XANES spectra of the tailings, Cr(III)- and Cr(VI)-ferrihydrite (FHY) references, and the (b) baseline subtracted pre-edge of the enclosed features (gray solid box) in (a). The dotted lines superimposed on the data in (b) denote linear combination fits (LCF) using Cr(III)-FHY, Cr(VI)-FHY, and Cr-hematite references (detailed in Text S2).

When analyzing Cr XAS patterns, there is a unique advantage in that the pre-edge features of Cr(III) and Cr(VI) are significantly different from each other (Figure S6). The X-ray absorption near edge structure (XANES) spectra of all tailings (Figures 4 and S7) displayed two weak pre-edge peaks at 5990 and 5993 eV, which are characteristic of spin-forbidden electron transitions in octahedrally coordinated Cr(III). The absence of the large and sharp pre-edge peak of tetrahedrally coordinated Cr(VI) at 5993 eV indicates that Cr predominantly exists as Cr(III) in the tailings, which is supported by the direct quantification through linear combination fitting (LCF) of the Cr XANES of the tailings (Figure 4b).

**Figure 5:**
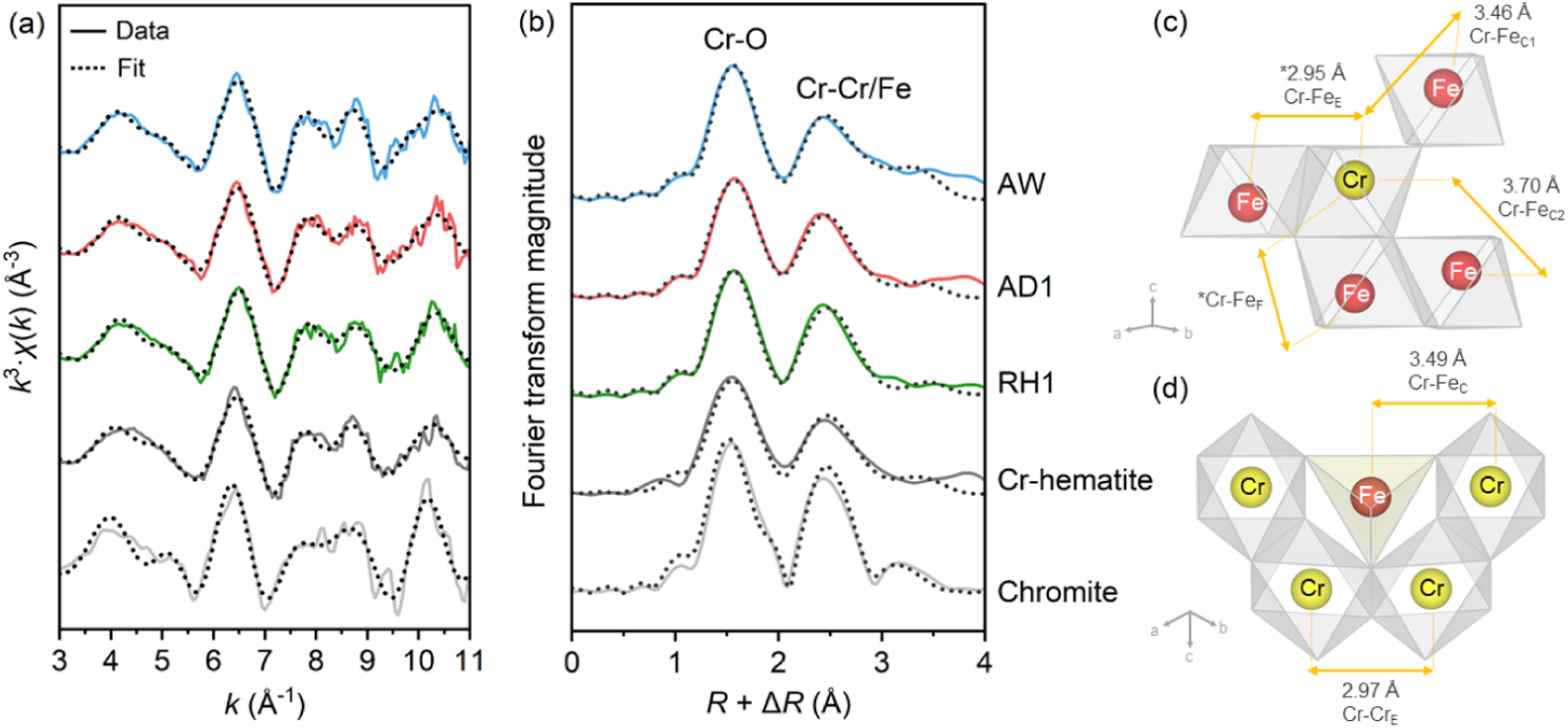
(a) Cr K-edge *k*^3^-weighted EXAFS spectra and the corresponding (b) Fourier transforms (FT) of tailings samples and reference minerals, Cr-hematite and chromite. Dotted lines superimposed on the solid lines denote shell-by-shell fits of the EXAFS data. Structural models show the Cr coordination environment of (c) Cr-hematite and (d) chromite including the Cr–Cr/Fe distances based on the best fit given in Table S6.

The extended X-ray absorption fine structure (EXAFS) spectra of the tailings show similar coordination environments (Figure 5a,b). Shell-by-shell fitting of the EXAFS spectra (Table S6) further confirmed the speciation of Cr, showing octahedrally coordinated Cr(III) with two distinct Cr–O shells at distances of 1.97–1.98 Å (axial) and 2.44–2.45 Å (equatorial). The Cr-hematite standard was also best fit with two Cr–O shells, suggesting Jahn–Teller distortion of Cr(III), consistent with the observations of Mn(III) in substituted Fe (oxyhydr)oxides reported by Scheinost et al.^41^ In fitting the closest neighboring shells, we refer to both Cr and Fe as “Cr/Fe” as their unique contributions to the EXAFS signal could not be distinguished because of their close atomic numbers. The best fit to the EXAFS data showed Cr–Cr/Fe shells at 2.94–2.95 Å, 3.43–3.46 Å, and 3.68–3.70 Å, matching the distances for the face-, edge-, and corner-shared octahedra of Cr-hematite and chromite (Figure 5c,d). Cr-hematite and chromite have very close first and second Cr–Cr/Fe distances and their contributions could be better distinguished through their coordination numbers. However, due to the high uncertainty of EXAFS-derived coordination numbers (20–25%),^42^ this proved to be difficult. As such, we combined the EXAFS fitting results with our mineral characterization data, and show that in the HPAL tailings, Cr was mainly structurally incorporated as Cr(III) in chromite and via substitution for octahedral Fe(III) in hematite.

### Partitioning and Mobility of Cr

**Figure 6:**
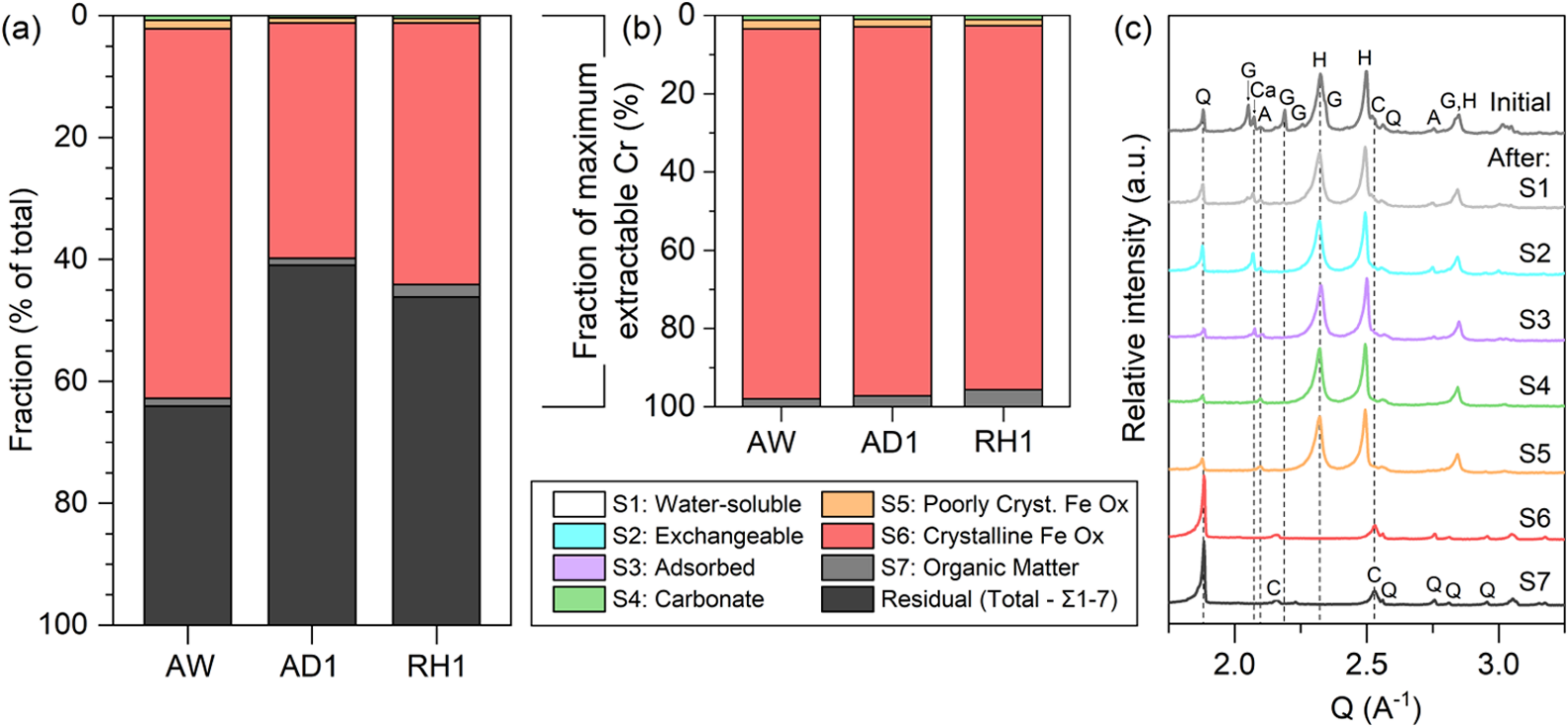
Sequential extraction data of selected samples showing the distribution of Cr fractions with respect to the (a) total Cr concentration and to the (b) maximum extractable Cr, which pertains to the sum of the nonresidual fractions (S1–S7). (c) XRD patterns of the residues of RH1 after each extraction step. See Figure S10 for the XRD patterns of AW and AD1 residues. A—alunite, C—chromite, Ca—calcite, G—gypsum, H—hematite, and Q—quartz.

Using sequential extraction, we gained a better understanding of the partitioning of solid-phase Cr in different mine tailings. This allowed us to assess the potential mobility of Cr species in the surrounding environment and relate these findings to the geochemical properties of the interacting waters. The first extraction step (Table S2) estimates water-soluble Cr through the interaction of the solids with ultrapure water. Chromium was not mobilized during this step (Figure 6a,b), confirming the absence of Cr in gypsum, a water-soluble mineral primarily dissolved in S1. XRD analysis of the residues (Figure 6c) validated the removal of gypsum in this step. The extraction and XRD results also corroborate the chemical composition of the water samples collected from the active tailings dam and from its outflow pond. These surface waters are characterized by circumneutral to slightly alkaline pH (7.5–8.0) and high calcium and sulfate contents (Table S4), reflecting the components of gypsum and the influence of the acid leaching–neutralization processes. More importantly, they also exhibit below detection total Cr (<14 μg L^–1^) and Cr(VI) (<10 μg L^–1^) concentrations. The next two extraction steps: exchangeable Cr (S2) and adsorbed Cr (S3) were also negligible in all tailings. Typically, exchangeable Cr represents weakly bound Cr that is susceptible to releases when changes in ionic strength occur, like for example during interaction with saline waters. On the other hand, adsorbed Cr represents Cr(VI) oxyanions likely to be desorbed from Fe (oxyhydr)oxides and other minerals in the presence of competing aqueous ions (e.g., phosphate in agricultural drainage).^10^ Our documented negligible amounts of Cr released during extraction steps S1 to S3 and in water samples from the active tailings dam demonstrate that Cr in the solid tailings is not easily mobilizable.

Upon further extraction steps, we show that minor proportions of the total Cr could be extracted from the carbonate (S4), poorly crystalline Fe (oxyhydr)oxide (S5), and organic matter (S7) fractions (Figure 6a). Below 1% of Cr was extracted by the acetate step (S4) that dissolves any carbonate remnants from the neutralization process (see also XRD patterns of residue in Figure 6c), and 0.7–1.4% were removed using 1 M HCl (S5) which dissolves poorly crystalline Fe (oxyhydr)oxides. The latter phases are often difficult to quantify by XRD as they would appear only as a background in samples with many crystalline phases. Both these fractions were cross-confirmed by the minor (<0.5 wt %) amount of Cr in the SEM-EDS analysis of the original limestones (Figure S8) used for neutralization, and by the low proportion of Fe (<1%) extracted in the poorly crystalline fraction (Figure S9). These two fractions are sensitive to changes in pH experienced, for example, during acid rain or interaction with organic acids.^43^ Furthermore, the oxidizable Cr fraction (S7), associated with organic matter, accounted for only 1.1 and 2.0 wt % of the tailings' Cr content.

The largest pools for Cr were associated with the crystalline Fe (oxyhydr)oxide (S6) and the recalcitrant residual fractions. These accounted for 39–61% and 37–60% of the total Cr concentration, respectively. Based on the XRD patterns of the residues (Figures 6c and S10), both hematite and alunite were dissolved during the 6 M HCl step (S6). To calculate the amount of Cr in each of these two mineral fractions, we combined our Rietveld analysis and SEM-EDS data of alunite (Text S4) and estimated that only 0.5–1.9% of the total Cr was associated with alunite while the majority was associated with hematite, supporting our TEM-EDX and XAS results (Figures 3 and 5). Comparing the various samples, the active wet sample (AW) exhibited the highest crystalline Fe (oxyhydr)oxide-bound Cr content (Figure 6a) matching its higher hematite proportion (Figure 1). The residual fraction that remained undissolved after sequential extraction was predominantly composed of chromite, as confirmed by XRD analysis of the final residue (Figure 6c) and the robust calibration of the sequential extraction procedure.^10^

## Discussion

### Fate of Cr during Tailings Formation

The studied tailings were characterized by Cr concentrations (Table S3) and mineral compositions (Figure 1) that were similar to HPAL residues of processed laterites in other prominent Ni-producing regions (Table S1), with one major difference being the presence of neutralization products (e.g., calcite, gypsum) in our samples. In the HPAL process, acid leaching mainly dissolves Fe (oxyhydr)oxides (e.g., goethite) and extracts Ni and Co together with Fe, Al, and Cr. Under HPAL conditions, Ni and Co remain in solution while the leached Fe and Al undergo rapid hydrolysis, resulting in the precipitation of oxides and sulfates,^22,23,39^ such as the hematite and alunite minerals observed in our tailings. Our SEM and TEM analyses (Figures 2 and 3a–f) indicated the association of Cr with hematite and alunite, suggesting the coprecipitation of Cr during their formation. Elemental mapping of FIB sections (Figure 3g–l) further revealed the distribution of Cr within the minerals, showing a higher concentration in hematite than alunite. In hematite, Cr could occur as adsorbed species of Cr(III) and Cr(VI), or readily substitute as Cr(III) for Fe(III) in its crystal structure due to their similar charge and ionic radii (octahedral radii: Cr(III) = 0.615 Å, Fe(III) = 0.645 Å).^44,45^ While there are very few studies that have reported the association between Cr and hematite in HPAL residues,^27^ their findings are limited to surface-level analyses (e.g., SEM) and do not address the mechanism of Cr binding. Our Cr K-edge XAS analysis (Figure 5) provides new direct evidence for the structural incorporation of Cr(III) into hematite in such mine tailings, underscoring its key role in sequestering Cr in less mobilizable fractions compared to adsorption, which involves more easily leachable species. Since residual Cr represents unleached relic chromite, while nonresidual fractions (S1–S7) correspond to acid-leached Cr that subsequently precipitated, our sequential extraction (Figure 6b) demonstrated that more than 90% of the nonresidual Cr was sequestered by hematite, mainly dissolved in step S6. Our extraction data (Figure 6), combined with XRD (Figure 1) and TEM results (Figure 3), also revealed minor proportions of Cr (up to 2%) associated with alunite. Previous studies have shown that Cr could be substituted in alunite as Cr(III) for Al(III) or as Cr(VI) in CrO_4_^2–^ for SO_4_^2–^.^28^ However, the minor abundance of alunite and associated Cr precluded determining the redox state of Cr, and more detailed examination is needed to explore this further.

Although some HPAL studies have suggested the oxidation of Cr(III) to Cr(VI) in the acid leach liquors,^22,26,28^ our EXAFS data highlight that Cr mainly occurs as Cr(III) in the tailings, with no trace of Cr(VI). The controlled oxidation–reduction potential in the HPAL autoclaves and the downstream processing of the acid leaching solutions such as sulfidization or the addition of H_2_S gas, as applied at Philippine sites (Figure S1b),^25^ may limit or prevent the oxidation of the original Cr(III) or promote the reduction of unwanted Cr(VI). In fact, controlling the redox potential through sulfur addition has been reported to inhibit Cr(VI) formation at a leaching plant in Moa Bay, Cuba.^22^

Overall, while acid leaching liberates Cr from laterite ores, it also creates ideal chemical pathways for the formation of minerals that capture Cr. The absence of Cr in minerals formed later in the HPAL process (e.g., gypsum) and mineral surfaces (i.e., exchangeable and adsorbed fractions) clearly support the fact that Cr has already been sequestered in the early phases of the process and remain immobilized during downstream operations and deposition of the tailings.

### Implications for Long-Term Behavior of Chromium

Despite the known environmental releases of Cr(VI) in laterite mine catchments,^4,6^ our work highlights the immobilization of Cr in tailings derived from HPAL-treated Ni–Co laterite ores. The studied mine tailings exhibit similarly elevated Cr concentrations (up to 1.5 wt %) as Ni–Co laterites (up to 7.0 wt %),^4,13,46^ compared with the average Cr composition of the upper crust (0.0035 wt %).^47^ However, they differ in that the laterites contain both Cr(III) and Cr(VI) species, whereas the mine tailings mainly consist of Cr(III). While natural weathering processes involving co-occurring oxidants (e.g., manganese oxides) promote the genesis of Cr(VI) in laterites,^16,48^ the controlled HPAL operation could inhibit its formation and favor the occurrence and sequestration of Cr(III) in poorly soluble minerals that limit its environmental mobility. Earlier sequential extraction steps indicated that Cr in the tailings remain largely immobile and does not dissolve into the aqueous phase upon interaction with water. This retention keeps dissolved Cr in interacting waters in the tailings dam to negligible levels, far below the global regulatory limit of 50 μg L^–1^. Given that even the outflow pond exhibits no measurable Cr, the downstream transport of Cr into receiving mine waters and ultimately into seawater is expected to be negligible.

This stability of Cr is largely controlled by the mineralogical forms in which Cr is bound. Relic chromite grains that remain undissolved after HPAL treatment (Figure 2a) and also after our sequential extraction (Figure 6c) represent an inert and weathering-resistant host for Cr. The secondary hematite, which sequesters the majority of Cr liberated by acid leaching, also proves to be a recalcitrant host for Cr. Hematite is one of the thermodynamically most stable Fe (oxyhydr)oxides and it is poorly soluble over a wide range of pH (2–14).^44,49,50^ As evidenced by our sequential extraction data, hematite remained unaffected by the interaction of tailings with solutions that represent several environmental conditions (e.g., rainfall, saltwater interaction, agricultural drainage) and only dissolved when exposed to a highly acidic and aggressive reagent. Moreover, the incorporation of Cr into Fe (oxyhydr)oxides like hematite has been found to increase mineral stability and resistance to dissolution^10,51^ since Cr forms stronger bonds with oxygen (e.g., Cr(III)–O = 24.5 kJ mol^–1^) compared to iron (e.g., Fe(III)–O = 23.7 kJ mol^–1^).^52^ The current lack of significant differences in speciation and partitioning of Cr between the active and rehabilitated tailings attest to the stability of Cr-hematite under various conditions (e.g., waterlogged versus dry), and even after long-term storage (>10 years) and rehabilitation. Although Cr-hematite may be stable under the present surface conditions of the tailings, reducing conditions could induce hematite dissolution^44,53^ and remobilization of Cr. Thus, it is crucial to monitor the stability of hematite where anoxic conditions are expected (e.g., subsurface, deep-sea tailings disposal). Moreover, because the rehabilitation of the tailings dam is being done through the use of vegetative cover and metal hyperaccumulating plants, it is also important to monitor rehabilitated tailings as organic acids from root exudates and siderophores have been reported to induce proton- and ligand-promoted dissolution of hematite.^54^

Prior to this study, the fate and long-term behavior of Cr in mine wastes generated from hydrometallurgical processing of Ni–Co laterite ores were not well understood. This research specifically enhances our fundamental understanding of Cr immobilization in HPAL operations that globally employ similar processes to extract Ni and Co from Fe (oxyhydr)oxide-rich laterite ores. This study also offers critical insights for the long-term management of mine tailings, which may, over time, undergo soil development processes and transition into technosols. Here, we presented evidence that the incorporation of Cr in resistant minerals like chromite and hematite, along with the very slow oxidation kinetics observed for Cr(III) in the environment,^55^ and particularly in the studied tailings, controls chromium's long-term stability and inhibits its transformation into the more mobile and toxic Cr(VI). Elucidating the fate of Cr and the mechanism of its immobilization is integral for a comprehensive risk assessment and effective environmental management of the growing HPAL waste volumes expected from the global energy transition.

## Supporting Information Available

Supporting materials and methods; HPAL overview; sampling sites; XRD patterns of mineral standards and tailings; particle size distribution; XANES and EXAFS fitting results; extraction protocol; elemental composition of the solid tailings and water samples; and supporting SEM micrographs and sequential extraction data (PDF)
