# Supplementary material for: Immobilization of Chromium by Iron Oxides in Nickel–Cobalt Laterite Mine Tailings
Source: Environ Sci Technol. 2025 Mar 14;59(11):5683–92. doi: 10.1021/acs.est.4c05383 (PMC11948461; doi:10.1021/acs.est.4c05383)
Supplement: Supplementary file 1 — es4c05383_si_001.pdf [file es4c05383_si_001.pdf]

Supporting information for

Environmental Science & Technology

Immobilization of chromium by iron oxides in nickel-cobalt laterite mine tailings

Ruth Esther Delina[1,2], Jeffrey Paulo H. Perez,[1*] Vladimir V. Roddatis,[1] Jessica A. Stammeier,[1] Damien Prieur[3,4], Andreas C. Scheinost[3,4], Mark M. Tan,[5] Jhonard John L. Garcia,[5] Carlo A. Arcilla,[5,6] Liane G. Benning[1,2]*

[1] GFZ Helmholtz Centre for Geosciences, Telegrafenberg, 14473 Potsdam, Germany

[2] Department of Earth Sciences, Freie Universität Berlin, 12249 Berlin, Germany

[3] The Rossendorf Beamline at ESRF, The European Synchrotron, CS 40220, 38043 Grenoble Cedex 9, France

[4] Institute of Resource Ecology, Helmholtz-Zentrum Dresden-Rossendorf, Bautzner Landstraβe 400, 01328 Dresden, Germany

[5] National Institute of Geological Sciences, University of the Philippines, Diliman, Quezon City 1101, Philippines

[6] Department of Science and Technology, Philippine Nuclear Research Institute, Diliman, Quezon City 1101, Philippines

*Corresponding authors: rdelina@gfz.de; jpperez@gfz.de

Summary: 17 pages, 4 text sections, 10 figures, 7 tables

Table of Contents

S1. Supporting Materials and Methods……S2

S2. Supplementary Figures……S4

S3. Supplementary Tables……S11

References……S16

S1. Supporting Materials and Methods

Text S1. Synthesis of Cr-ferrihydrites

Following the method of Schwertmann and Cornell[1], Cr(III)- and Cr(VI)-bearing ferrihydrites containing 1 wt.% Cr were synthesized by dropwise addition (2.9 mL min^{-1}) of 1 M NaOH to 150 mL of mixed solution of 195.6 mM $Fe(NO_3)_3·9H_2O$ and 4.4 mM $Cr(NO_3)_3·9H_2O$ or K_2CrO_4 until pH 7 is reached. The solids were separated by centrifugation (10,052*g*, 10 min), washed eight times using Milli-Q water (~18.2 MΩ·cm) to remove remaining electrolytes, and freeze-dried. **Figure S3** shows the diffractogram of the mineral standards used in this study confirming the purity of the Cr-ferrihydrites and other references.

Text S2. Cr K-edge XANES data analysis

The redox state of Cr in the tailings samples was determined through linear combination fitting (LCF) of the pre-edge region (5986-5997 eV) extracted from the normalized XANES spectra by baseline subtraction using the XANES dactyloscope software.[2] The LCF was performed using the ATHENA[3] software and Cr(III)-ferrihydrite, Cr(VI)-ferrihydrite, and Cr-hematite as references. Tetrahedral Cr(VI) shows a strong absorption peak at 5993 eV while octahedral Cr(III) has two weak peaks at 5990 and 5993 eV.[4] Considering the significant partitioning of Cr in hematite based on the sequential extraction data, Cr-hematite was added as reference as it shows a relatively more intense pre-edge peak in the 5991-5993 eV range compared to Cr(III)-ferrihydrite (**Figure S6**). Such feature can be explained by the structural distortion of the octahedral environment of Cr(III) in hematite structure.[5] By adding Cr-hematite in the LCF, we avoid identifying peaks around 5993 eV as Cr(VI). This technique has also been applied by previous studies.[5]

Text S3. Cr K-edge EXAFS fitting

EXAFS fitting was performed following the shell-by-shell approach described in Delina et al.[6]. In brief, Fourier transforms filtered from the k^3-weighted EXAFS data were fit over 1.2 to 3.8 Å R-space in SIXpack[7] using the algorithms derived from IFEFFIT.[8] The fitting was done by defining shells of

neighboring atoms and then iterating the coordination numbers (CN), distances (R), and the mean squared atomic displacement parameter or Debye-Waller factors (σ^2) while the passive electron reduction parameter, S_0^2, was constrained to a value of 0.7. In this approach, the CN of the reference minerals (i.e., chromite, Cr-hematite) were fixed according to literature values to decrease the degrees of freedom and fit-derived standard errors from highly correlated parameters (e.g., CN and σ^2). The coordination environment of Cr in the references were used to constrain the σ^2 values in the tailings' spectra, allowing CN to adjust in the fit. The goodness of fit represented by the R-factor: $R = \sum_i(data_i - fit_i)^2/\sum_i(data_i)^2$ was used to assess the quality of the spectral fits. An R-factor below 0.05 signifies a reasonable fit.[9] In addition, the F-test described in Downward et al.[10] was performed to statistically assess if a change in the model (e.g., addition of a shell) improves the fit. A significantly better fit yields a confidence level, α, that is greater than 67%. A sample F-test is given in **Table S7**.

Text S4. Estimation of mineral fraction using Rietveld and SEM-EDS data

To estimate the fraction of Cr (% of total concentration) associated with certain minerals, we use the following formula:

$$Cr\ fraction\ \% = \frac{Phase\ composition\ x\ C_{SEM}}{C_{Total}}\ x\ 100$$

where,

$Phase\ composition$ = Rietveld calculated phase composition

C_{SEM} = median Cr concentration of mineral measured using SEM-EDS

C_{Total} = total Cr concentration of tailings measured using ICP-OES (Table S3)

In the case of alunite that comprises 0.9-3.5% of the mineral composition of the tailings and is characterized by a median Cr concentration (*n* = 15) of 0.7 ± 0.1 wt.% based on SEM-EDS analysis, the calculated Cr fraction in alunite ranges from 0.5% ± 0.1% to 1.9% ± 0.3% of the total Cr.

S2. Supplementary Figures

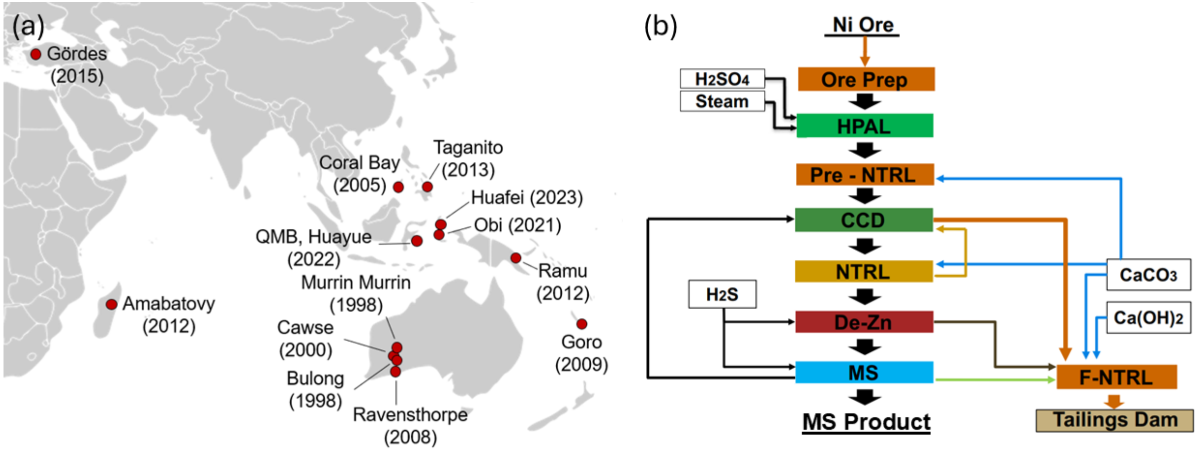

Figure S1. (a) HPAL operations concentrated in Asia, Australia and Oceania, with the start of operations (year) noted in parentheses. Data sourced from Gultom and Sianipar[11] and Stanković et al.[12]. Other notable HPAL plants not depicted on the map include Moa Bay in Cuba, which began operations in 1959. (b) Example HPAL process workflow for Ni laterite ores (Shibayama et al.[13]). Adapted with permission from 13. Copyright © 2016 Elsevier Ltd. During HPAL processing, Ni and Co are leached using sulfuric acid under high temperature (230-270°C) and pressure (3.3-5.5 MPa) conditions followed by several processes including multi-stage neutralization (NTRL) using limestone ($CaCO_3$). Counter-current decantation (CCD) is conducted to separate the Ni- and Co-rich solution (also known as pregnant leach solution) and the barren leach residue. Downstream processing of the leach solutions can be done through mixed sulfide precipitation (MS) by the addition of H_2S or in other plants, mixed hydroxide precipitation by the addition of caustic magnesia (MgO) or caustic soda (NaOH). After the final treatment (F-NTRL) with limestone and slaked lime ($Ca(OH)_2$), the neutralized slurry is discharged to the tailings dam. Please refer to Shibayama et al.[13] and other reviews (e.g., Whittington and Muir[14]) for a detailed description of the full HPAL process.

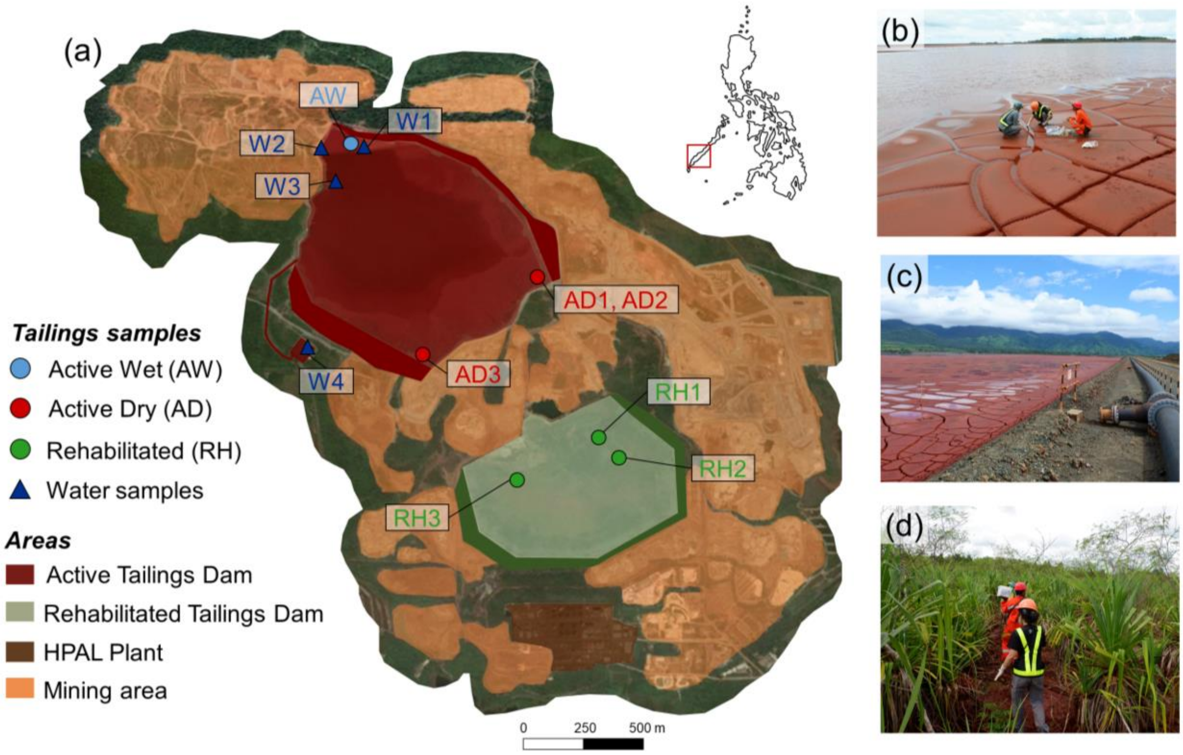

Figure S2. (a) Map of the study area showing the sampling sites, with photographs of the (b) waterlogged and (c) dry areas of the active tailings dam, and a (d) lush green revegetated area of the rehabilitated tailings dam. The average human height of 1.6 m (b,d) and a meter rod (c) were used as scale. Map data © 2023 Google.

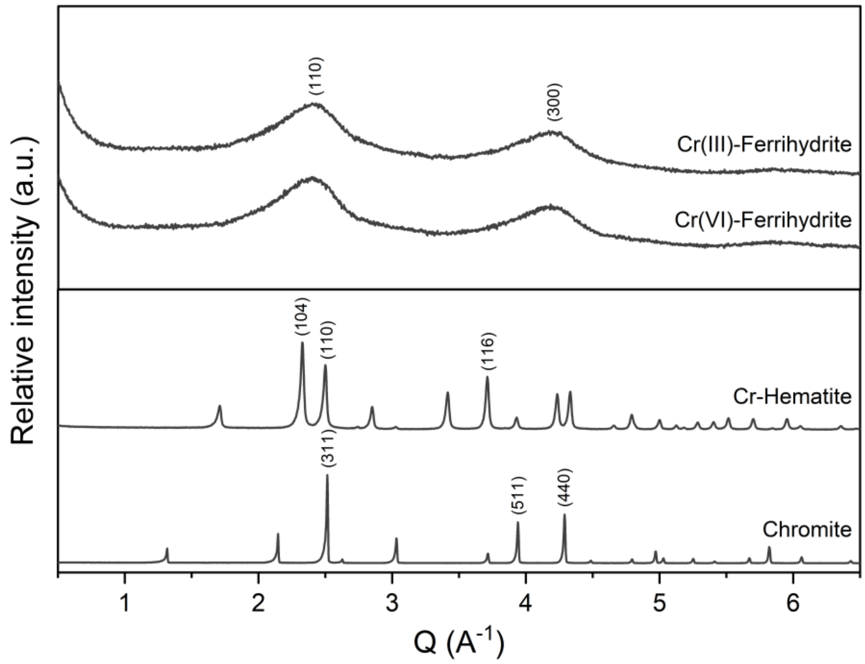

Figure S3. Powder X-ray diffractograms of mineral standards with indexed main reflections.

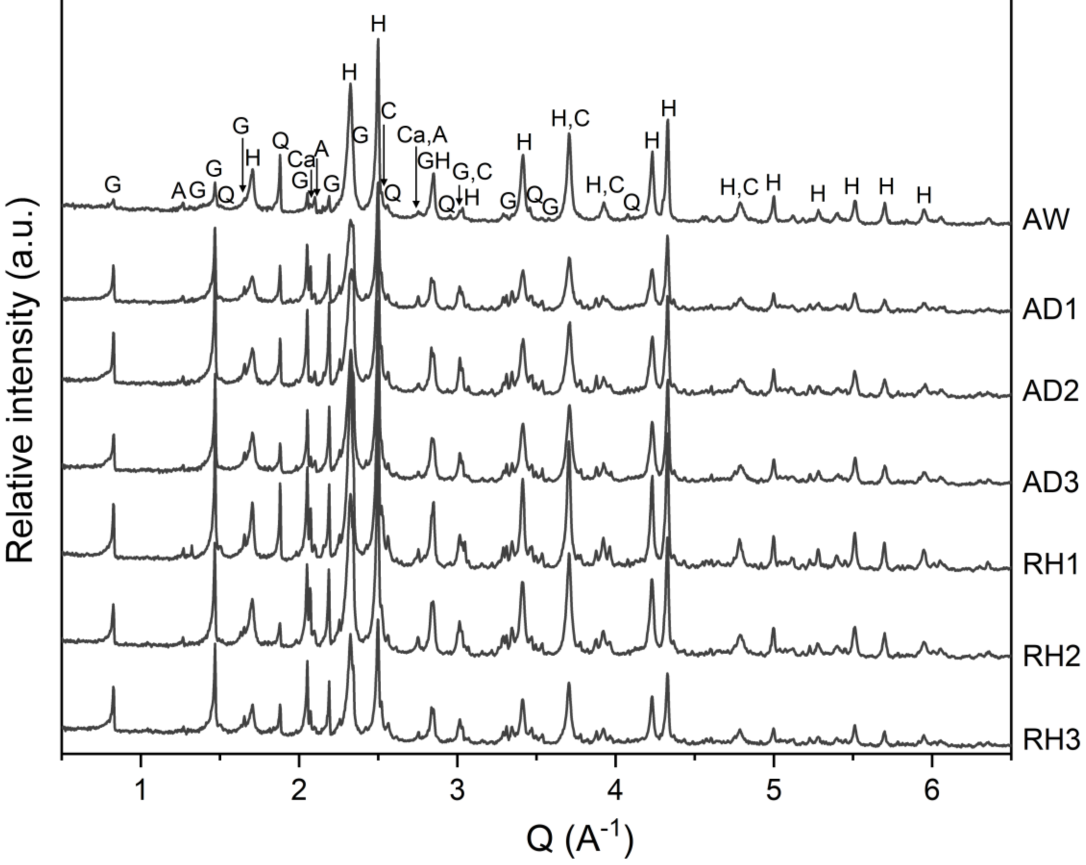

Figure S4. Powder X-ray diffraction patterns of the active wet (AW), active dry (AD) and rehabilitated (RH) tailings and their corresponding mineral composition: A – alunite, C – chromite, Ca – calcite, G – gypsum, H – hematite, Q – quartz

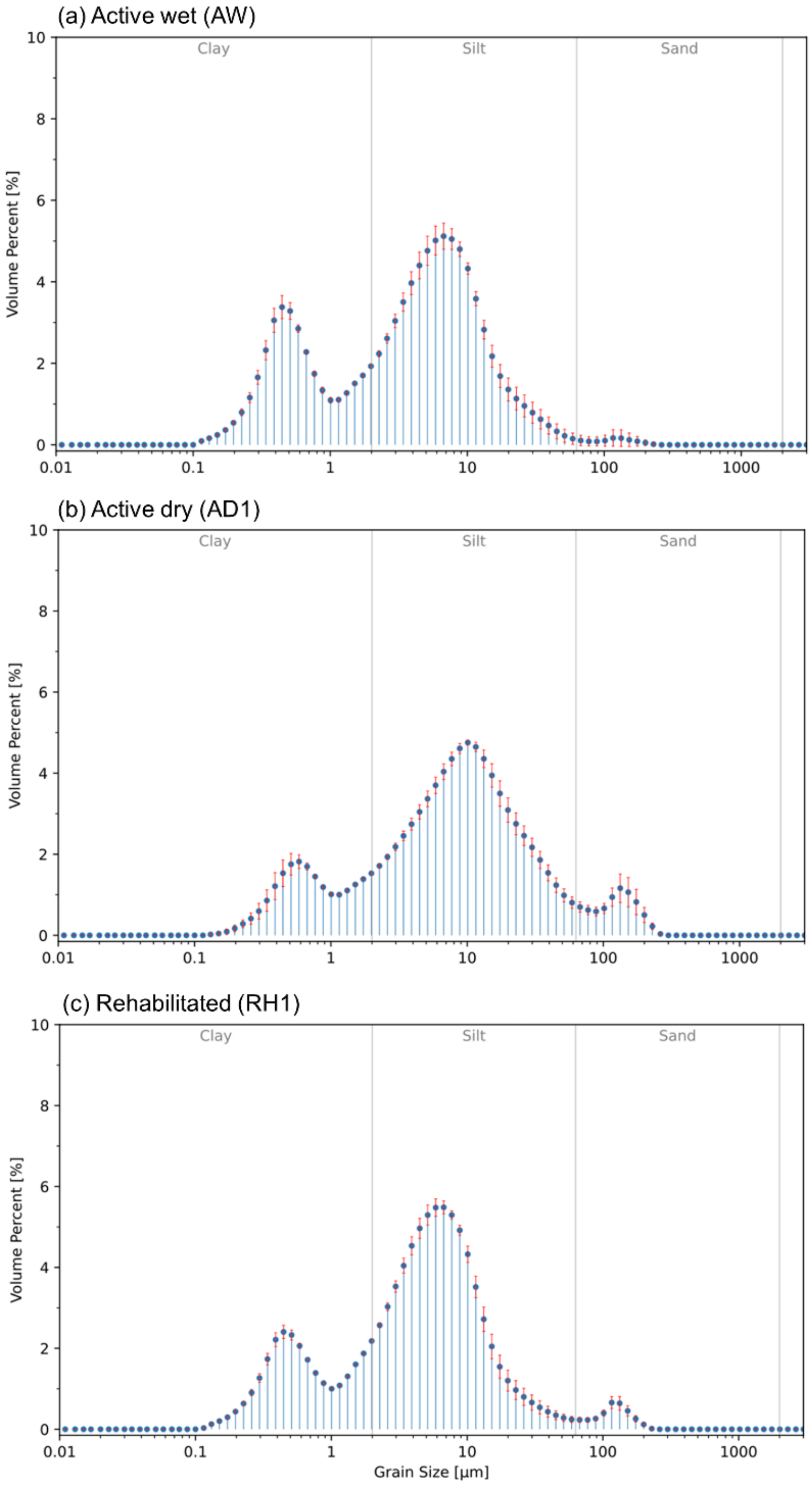

Figure S5. Particle size distributions of representative samples: (a) wet and (b) dry tailings from the active tailings dam, and (c) rehabilitated tailings. The blue dots and red whisker lines indicate the arithmetic mean and 95% confidence interval, respectively. It is important to note that the larger grain size fraction (>80 µm), corresponding mainly to water-soluble gypsum crystals, may be slightly affected by the sample pre-treatment (i.e., ultrasonication with water).

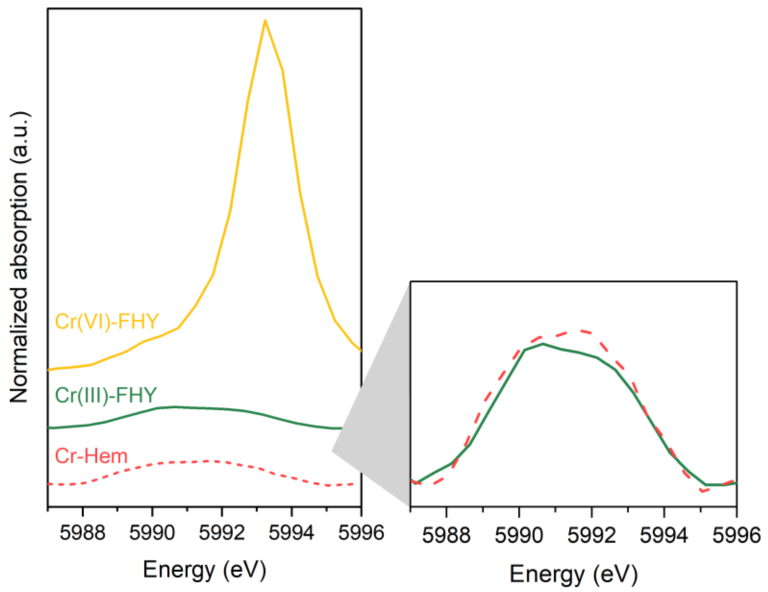

Figure S6. Comparison of the Cr K-edge XANES pre-edge collected from the Cr (III)- and Cr(VI)-ferrihydrites (FHY) and Cr(III)-hematite (Hem).

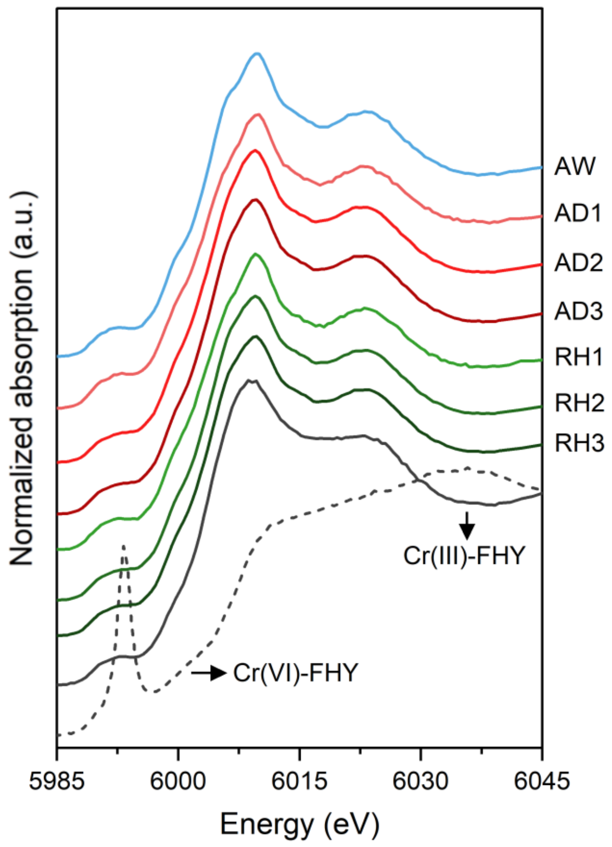

Figure S7. Normalized Cr K-edge XANES spectra of all tailings samples (AW – active wet, AD – active dry, RH – rehabilitated), and Cr(III)- and Cr(VI)-bearing ferrihydrites.

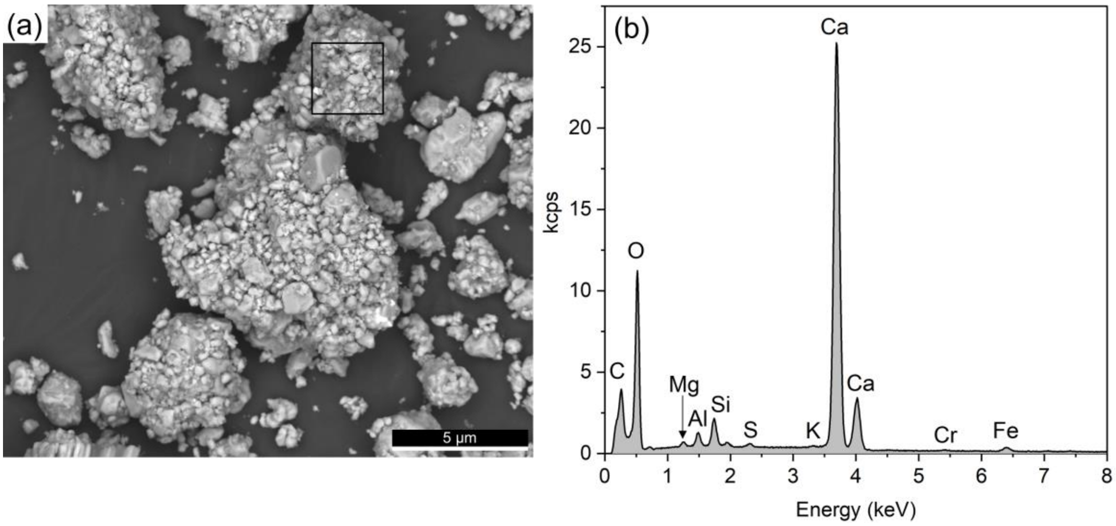

Figure S8. (a) SEM image of a limestone used for neutralization in the HPAL process and the (b) corresponding EDS spectrum of the boxed area in (a) showing a minor amount of Cr (<0.5 wt.%).

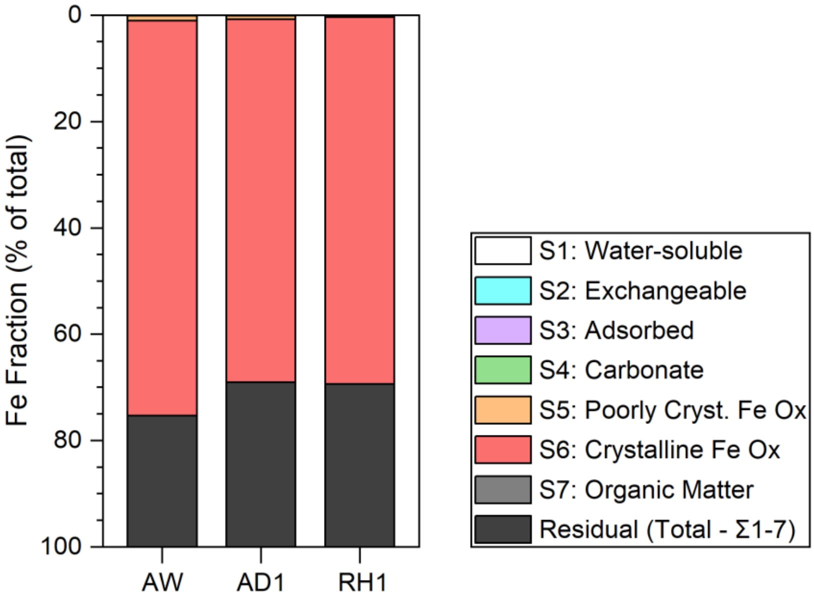

Figure S9. Fe partitioning in the representative samples from the active wet (AW), active dry (AD1), and rehabilitated (RH1) tailings. Ox – (oxyhydr)oxide

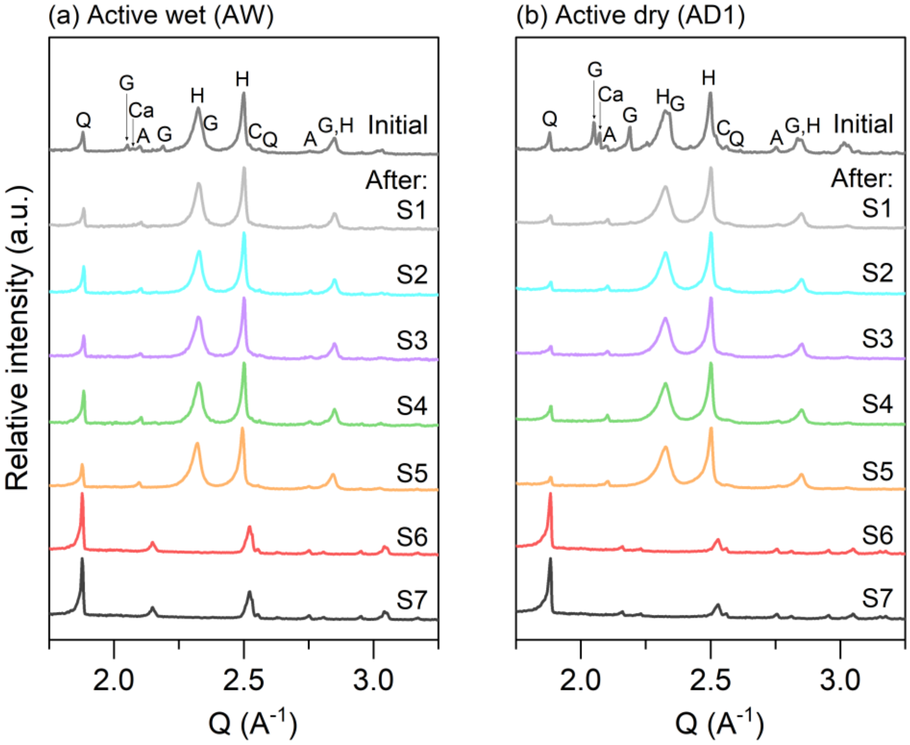

Figure S10. Powder XRD patterns of the residues of tailings samples AW and AD1 after each sequential extraction step (S1 – Water-soluble, S2 – Exchangeable, S3 – Adsorbed, S4 – Carbonate, S5 – Poorly crystalline Fe (oxyhydr)oxide, S6 – Crystalline Fe (oxyhydr)oxide, S7 – Organic matter). A – alunite, C – chromite, Ca – calcite, G – gypsum, H – hematite, Q – quartz

S3. Supplementary Tables

Table S1. Summary of selected literature data on HPAL residues from processing plants and laboratory-scale tests prior to neutralization.

Source	Origin	Composition (wt.%)			Mineral composition	Reference
		Fe	Cr	Al		
HPAL plant	New Caledonia	53.7	1.28	1.68	Hematite, quartz, natroalunite, Mg-silicate, chromite	Ang et al.[15]
HPAL plant	Indonesia	38.6	0.94	2.97	-	Gultom et al.[11]
Lab-scale tests	Australia	11.7-38.2	0.5-1.2	0.7-4.0	Hematite, quartz, alunite/jarosite, amorphous silica	Whittington et al.[16]
Lab-scale tests	Turkey	36.5	1.13	0.92	Hematite, quartz, alunite	Önal and Topkaya[17]
Lab-scale tests	Turkey	-	-	-	Hematite, quartz, goethite, anhydrite	Ucyildiz and Girgin[18]
Lab-scale tests	Turkey	-	-	-	Hematite, quartz	Kaya and Topkaya[19]

Table S2. Sequential extraction procedure adapted for the tailings. A solid-to-liquid ratio of 1:100 was applied for all except for organic Cr where 1:20 was employed.

Step	Target Fraction	Extractant	Conditions	References
S1	Water-soluble	Milli-Q water (~18.2 MΩ·cm)	1 h, 25 °C	Dold[20]
S2	Exchangeable	1 M $MgCl_2$	1 h, 25 °C	Claff et al.[21]
S3	Adsorbed	0.01 M $NH_4H_2PO_4$ (pH 8)	16 h, 25 °C	Delina et al.[6]
S4	Carbonate	1 M NaOAc (pH 4.5)	5 h, 25 °C	Delina et al.[6]
S5	Poorly crystalline Fe (oxyhydr)oxides	1 M HCl	8 h, 25 °C	Delina et al.[6]
S6	Crystalline Fe (oxyhydr)oxides	6 M HCl	24 h, 75 °C	Delina et al.[6]
S7	Organic matter	5% NaOCl (pH 8.5)	30 min, Boiling	Delina et al.[6]

Note: For each extraction step, the extractant was added to the powdered sample in an acid-cleaned centrifuged tube, and mixed using a temperature-controlled orbital shaker at 250 rpm. Phase separation was done by centrifugation at 10,052*g* for 10 min. Each step was followed by an intermediate washing step with Milli-Q water (~18.2 MΩ·cm). The solid residue was freeze-dried while the extracts and washes were acidified with concentrated HCl (Aristar® VWR) and analyzed by ICP-OES.

Table S3. Major element composition of the tailings reported as oxide (wt.%) following standard methods.[22]

	Active Wet	Active Dry			Rehabilitated		
	AW	AD1	AD2	AD3	RH1	RH2	RH3
Al_2O_3	3.99	3.71	3.68	2.84	3.18	2.67	3.02
CaO	3.68	10.5	10.1	9.08	12.0	10.7	11.6
Cr_2O_3	1.88	2.07	2.17	1.74	1.80	1.50	1.50
Fe_2O_3	53.2	43.4	44.4	41.2	48.9	40.8	42.5
MgO	3.41	2.91	2.84	2.43	1.69	1.51	2.14
MnO	0.84	0.61	0.57	0.58	0.69	0.63	0.72
SiO_2	17.6	15.2	15.1	10.8	8.85	7.85	11.1
TiO_2	0.11	0.11	0.11	0.09	0.07	0.09	0.06

Table S4. Summary of the physicochemical characteristics and major ion composition of the water samples (see **Figure S2**) and the saturation indices of mineral phases calculated from the water chemistry. EC – electric conductivity, DO – dissolved oxygen

Sample		Active Dam		Outflow pond
	W1	W2	W3	W4
Parameters				
Temperature (°C)	30.1	31.5	32.7	30.8
pH	7.8	7.8	8.0	7.5
Eh (mV)	366	372	390	397
EC (mS cm^{-1})	3.74	3.76	3.65	3.78
DO (mg L^{-1})	6.3	6.3	7.1	6.8
Major ions (mg L^{-1})				
Ca^{2+}	477	468	465	398
K^+	0.33	0.36	0.30	0.21
Mg^{2+}	237	225	221	176
Na^+	417	405	399	299
Cl^-	92.9	93.9	92.1	93.1
HCO_3^-	75.1	72.6	72.4	81.9
NO_3^-	*bdl*	693	*bdl*	*bdl*
SO_4^{2-}	1955	2409	1482	1968
Mineral saturation indices (SI)				
Anhydrite ($CaSO_4$)	-0.46	-0.40	-0.54	-0.49
Brucite ($Mg(OH)_2$)	-3.32	-3.38	-2.91	-4.06
Calcite ($CaCO_3$)	0.57	0.51	0.79	0.23
Dolomite ($CaMg(CO_3)_2$)	2.02	1.89	2.43	1.28
Epsomite ($MgSO_4{\cdot}7H_2O$)	-2.94	-2.90	-3.05	-3.03
Gypsum ($CaSO_4{\cdot}2H_2O$)	-0.28	-0.23	-0.37	-0.32
Halite (NaCl)	-6.13	-6.14	-6.14	-6.26
Magnesite ($MgCO_3$)	-0.18	-0.25	0.01	-0.58

bdl – below detection limit

Table S5. Elemental composition (in wt. %) from SEM-EDS point analysis of chromite, hematite, and alunite grains found in the tailings balanced to 100% with O.

Mineral types	Point	Chemical composition (wt.%)										
		C*	Na	Mg	Al	Si	S	Ca	Cr	Mn	Fe	O
Chromite	1	4.4	0.3	4.5	4.8	0.1	0.1	0.1	34.7	0.1	10.4	40.1
	2	*bdl*	*bdl*	2.9	4.5	0.2	*bdl*	*bdl*	43.1	*bdl*	16.1	33.0
	3	3.2	*bdl*	5.5	8.6	0.5	0.3	0.4	27.8	0.5	13.4	39.6
	4	1.0	*bdl*	2.4	5.1	0.1	*bdl*	*bdl*	38.9	0.7	15.8	34.3
	5	0.0	*bdl*	1.7	2.8	0.1	*bdl*	*bdl*	44.9	0.9	17.2	32.2
	6	8.3	*bdl*	4.4	4.3	0.8	0.3	0.1	22.8	0.4	12.3	46.2
	7	2.6	*bdl*	6.3	10.5	0.3	0.1	0.2	29.4	0.4	10.8	39.4
	8	0.2	0.1	5.7	4.6	0.4	0.1	0.1	41.0	*bdl*	13.9	33.9
	9	1.9	0.1	1.0	1.7	0.2	0.1	0.8	43.2	*bdl*	14.6	35.0
	10	1.5	*bdl*	0.8	1.9	0.2	*bdl*	0.5	41.9	*bdl*	17.9	34.2
	Min.	*bdl*	*bdl*	0.8	1.7	0.1	*bdl*	*bdl*	22.8	*bdl*	10.4	32.2
	Max.	8.3	0.3	6.3	10.5	0.8	0.3	0.8	44.9	0.9	17.9	46.2
	Median	1.9	0.1	3.7	4.6	0.2	0.1	0.2	40.0	0.5	14.3	34.7
	**Ave.**	**2.6**	**0.2**	**3.5**	**4.9**	**0.3**	**0.2**	**0.3**	**36.8**	**0.5**	**14.2**	**36.8**
Hematite	1	0.5	*bdl*	0.8	1.4	2.4	0.6	0.2	1.0	0.7	59.3	33.0
	2	0.2	*bdl*	0.8	1.4	2.4	1.0	0.4	1.0	0.6	59.4	32.8
	3	2.7	*bdl*	0.2	1.5	2.9	0.6	0.2	0.9	0.4	53.8	36.8
	4	0.5	*bdl*	0.2	1.0	1.5	0.6	0.2	1.0	0.7	61.9	32.3
	5	10.7	0.2	0.4	1.8	2.3	0.3	0.2	0.5	0.3	33.8	48.6
	6	*bdl*	*bdl*	2.9	2.7	4.7	1.2	1.7	1.0	0.9	50.3	34.5
	7	*bdl*	*bdl*	1.2	1.3	3.2	0.8	0.9	1.2	1.5	56.4	32.8
	8	4.4	0.7	1.6	2.1	3.1	0.4	0.3	0.9	0.6	46.3	39.6
	9	8.8	*bdl*	0.9	1.2	1.4	0.6	*bdl*	0.9	*bdl*	40.6	45.5
	10	3.7	*bdl*	0.4	0.8	1.4	0.7	*bdl*	1.3	*bdl*	54.5	37.4
	11	1.8	*bdl*	0.9	1.6	3.0	0.7	0.6	0.9	0.4	54.5	35.4
	12	8.2	*bdl*	3.5	2.5	3.7	0.8	0.2	0.5	0.5	33.7	46.5
	13	8.3	0.7	0.7	1.4	1.5	0.6	0.2	0.6	0.5	40.3	44.7
	14	5.7	*bdl*	0.1	0.7	1.5	0.5	0.4	0.7	0.5	49.2	40.3
	15	8.6	*bdl*	1.2	1.7	1.9	0.6	0.5	0.6	0.4	38.7	45.6
	Min.	*bdl*	*bdl*	0.1	0.7	1.4	0.3	*bdl*	0.5	*bdl*	33.7	32.3
	Max.	10.7	0.7	3.5	2.7	4.7	1.2	1.7	1.3	1.5	61.9	48.6
	Median	4.4	0.7	0.8	1.4	2.4	0.6	0.3	0.9	0.5	50.3	37.4
	**Ave.**	**4.9**	**0.5**	**1.1**	**1.5**	**2.5**	**0.7**	**0.5**	**0.9**	**0.6**	**48.8**	**39.1**
Alunite	1	13.6	*bdl*	0.7	11.8	0.6	6.8	*bdl*	0.3	5.5	*bdl*	60.6
	2	9.9	0.6	0.3	13.3	0.6	8.2	0.5	0.5	*bdl*	9.9	56.2
	3	*bdl*	0.2	*bdl*	19.4	0.4	14.5	0.3	0.8	0.1	16.8	47.2
	4	7.9	1.3	0.2	12.5	0.4	8.8	*bdl*	0.6	0.1	15.1	53.1
	5	14.5	*bdl*	0.7	10.4	0.7	6.1	*bdl*	0.3	*bdl*	6.1	61.1
	6	5.7	0.0	0.2	17.1	0.7	12.0	0.2	0.6	*bdl*	9.4	53.9
	7	4.1	0.6	0.7	15.7	1.2	10.5	0.3	0.8	0.1	15.7	50.1
	8	*bdl*	1.3	0.6	19.0	1.2	13.7	*bdl*	0.9	*bdl*	16.3	47.0
	9	6.8	1.0	0.5	14.3	0.8	10.7	*bdl*	0.8	*bdl*	11.5	53.7
	10	0.0	1.2	*bdl*	22.3	0.4	16.0	*bdl*	0.9	*bdl*	9.7	49.4
	11	9.2	0.1	0.5	13.0	1.2	7.8	0.1	0.5	*bdl*	12.4	55.2
	12	0.0	0.3	0.3	15.7	0.7	14.1	0.2	1.1	0.2	20.7	46.0
	13	0.0	0.7	*bdl*	19.7	0.3	15.7	0.1	0.7	*bdl*	13.6	48.0
	14	2.7	0.5	0.5	15.9	1.1	13.1	0.7	0.7	*bdl*	14.7	49.7
	15	15.6	0.2	0.3	9.8	0.4	5.1	0.2	0.3	*bdl*	6.6	61.6
	Min.	*bdl*	*bdl*	*bdl*	9.8	0.3	5.1	0.1	0.3	*bdl*	*bdl*	46.0
	Max.	15.6	1.3	0.7	22.3	1.2	16.0	0.7	1.1	5.5	20.7	61.6
	Median	6.8	0.6	0.5	15.7	0.7	10.7	0.2	0.7	0.1	13.0	53.1
	**Ave.**	**6.9**	**0.6**	**0.5**	**15.3**	**0.7**	**10.9**	**0.3**	**0.7**	**1.2**	**12.8**	**52.9**

*C values may be affected by C deposition in the SEM chamber during analyses but they were left in to account for the presence of C signal from C-bearing minerals (e.g., calcite). *bdl* – below detection limit

Table S6. Cr K-edge EXAFS fitting results summarizing the local coordination environment around a central Cr atom for the reference minerals and the tailings.[a]

Sample	Atomic pair	CN	R (Å)	σ^2 (Å^2)	ΔE_0 (eV)	*R*-value
AW - Active (wet)[b]	Cr-O_1	4.9 (0.5)	1.98 (0.02)	*0.001*	-4.6 (3.7)	0.017
	Cr-O_2	2.5 (0.9)	2.44 (0.04)	$\sigma_{Cr\text{-}O1}$		
	Cr-Cr/Fe_1[c]	3.8 (0.7)	2.95 (0.03)	*0.005*		
	Cr-Cr/Fe_2	5.2 (2.6)	3.43 (0.04)	*0.008*		
	Cr-Cr/Fe_3	8.4 (4.2)	3.69 (0.04)	*0.011*		
AD1 - Active (dry)	Cr-O_1	4.0 (0.5)	1.97 (0.02)	*0.001*	-4.6 (4.1)	0.020
	Cr-O_2	2.7 (0.8)	2.44 (0.04)	$\sigma_{Cr\text{-}O1}$		
	Cr-Cr/Fe_1	3.8 (0.6)	2.94 (0.03)	*0.005*		
	Cr-Cr/Fe_2	4.0 (2.6)	3.44 (0.05)	*0.008*		
	Cr-Cr/Fe_3	5.7 (4.4)	3.68 (0.05)	*0.011*		
RH1 - Rehabilitated	Cr-O_1	4.2 (0.4)	1.97 (0.01)	*0.001*	-3.2 (3.0)	0.015
	Cr-O_2	2.1 (0.6)	2.45 (0.03)	$\sigma_{Cr\text{-}O1}$		
	Cr-Cr/Fe_1	4.1 (0.5)	2.94 (0.02)	*0.005*		
	Cr-Cr/Fe_2	3.6 (2.1)	3.46 (0.04)	*0.008*		
	Cr-Cr/Fe_3	4.8 (3.6)	3.70 (0.05)	*0.011*		
Cr-hematite	Cr-O_1	*4*	1.97 (0.02)	0.001 (0.001)	-5.7 (4.2)	0.028
	Cr-O_2	*2*	2.43 (0.04)	*0.001*		
	Cr-Fe_{F+E}[d]	*4*	2.95 (0.03)	0.005 (0.001)		
	Cr-Fe_{C1}	*3*	3.46 (0.07)	0.008 (0.006)		
	Cr-Fe_{C2}	*6*	3.70 (0.06)	0.011 (0.006)		
Chromite	Cr-O	*6*	1.98 (0.01)	0.0009 (0.0006)	-7.1 (1.7)	0.039
	Cr-Cr_E	*6*	2.97 (0.01)	0.005 (0.001)		
	Cr-Fe_C	*4*	3.49 (0.03)	0.008 (0.003)		

[a] CN, coordination number; R, interatomic distance; σ^2, mean-squared atomic displacement; ΔE_0, change in threshold energy; and *R,* "goodness of fit" factor. The passive electron reduction factor (S_0^2) was fixed at 0.7. Numbers in parenthesis are fit-determined standard errors. Constrained parameters appear in italics and without a parenthesis. All fits were carried out from 1.2 to 3.8 Å in *R*-space

[b] F-test done for the Cr-Fe paths of AW are summarized in Table S7 as an example

[c] Cr/Fe – pertains to Cr and Fe as their unique contributions to the EXAFS signal could not be distinguished due to their close atomic numbers

[d] F – face, E – edge, C – corner sharing

Table S7. F-test results of additional scattering paths used in the Cr K-edge EXAFS fitting of AW.[a]

Fit	Path	CN	*R* (Å)	σ^2 (Å^2)	ΔE_0 (eV)	χ^2	χ_v^2	R-factor	N_{IDP}	N_{VARYS}	DF	Confidence level α (%)
Cr-O only	Cr-O_1	4.9 (1.2)	2.00 (0.04)	*0.001*	0.06 (8.6)	2348	270	0.3287	13.7	5	8.7	
	Cr-O_2	2.5 (2.2)	2.49 (0.08)	$\sigma_{Cr\text{-}O1}$								
Addition of Cr-Cr/Fe_1[b]	Cr-O_1	5.0 (0.7)	1.98 (0.02)	*0.001*	-2.7 (4.2)	4628	81	0.0646	13.7	8	5.7	98%
	Cr-O_2	2.2 (1.2)	2.46 (0.05)	$\sigma_{Cr\text{-}O1}$								
	Cr-Cr/Fe_1	6.8 (4.1)	2.97 (0.03)	0.009 (0.005)								
Addition of Cr-Cr/Fe_2	Cr-O_1	4.9 (0.7)	1.97 (0.03)	*0.001*	-6.2 (6.0)	3498	74	0.0488	13.7	9	4.7	72%
	Cr-O_2	2.7 (1.2)	2.43 (0.05)	$\sigma_{Cr\text{-}O1}$								
	Cr- Cr/Fe_1	3.5 (0.9)	2.94 (0.04)	*0.005*								
	Cr- Cr/Fe_2	3.1 (2.1)	3.38 (0.06)	*0.008*								
Addition of Cr-Cr/Fe_3	Cr-O_1	4.9 (0.5)	1.98 (0.02)	*0.001*	-4.6 (3.7)	123	45	0.0171	13.7	11	2.7	76%
	Cr-O_2	2.5 (0.9)	2.44 (0.04)	$\sigma_{Cr\text{-}O1}$								
	Cr- Cr/Fe_1	3.8 (0.7)	2.95 (0.03)	*0.005*								
	Cr- Cr/Fe_2	5.2 (2.6)	3.43 (0.04)	*0.008*								
	Cr- Cr/Fe_3	8.4 (4.2)	3.69 (0.04)	*0.011*								

[a] CN, coordination number; R, interatomic distance; σ^2, mean-squared atomic displacement; ΔE_0, change in threshold energy; χ^2, chi-square; χ_v^2, reduced chi-square; *R,* "goodness of fit" factor; N_{IDP}, number of independent points; N_{VARYS}, number of variables; DF, degrees of freedom; and Confidence level α, statistical validity that added scattering path improved the fit based on the F-test. Numbers in parenthesis are fit-determined standard errors. Constrained parameters appear in italics and without a parenthesis

[b] Cr/Fe – pertains to Cr and Fe as their unique contributions to the EXAFS signal could not be distinguished due to their close atomic numbers

References

(1) Schwertmann, U.; Cornell, R. M. *Iron Oxides in the Laboratory: Preparation and Characterization*. Wiley: Germany, 2000, p 204.
(2) Klementiev, K. V. *XANES dactyloscope for Windows, freeware: www.desy.de/~klmn/xanda.html*. 2006.
(3) Ravel, B.; Newville, M. ATHENA, ARTEMIS, HEPHAESTUS: data analysis for X-ray absorption spectroscopy using IFEFFIT. *Journal of Synchrotron Radiation* **2005**, *12*, 537-541.
(4) Peterson, M. L.; Brown, G. E.; Parks, G. A.; Stein, C. L. Differential redox and sorption of Cr (III/VI) on natural silicate and oxide minerals: EXAFS and XANES results. *Geochimica et Cosmochimica Acta* **1997**, *61* (16), 3399-3412.
(5) Fandeur, D.; Juillot, F.; Morin, G.; Olivi, L.; Cognigni, A.; Webb, S. M.; Ambrosi, J.-P.; Fritsch, E.; Guyot, F.; Brown, J. G. E. XANES Evidence for Oxidation of Cr(III) to Cr(VI) by Mn-Oxides in a Lateritic Regolith Developed on Serpentinized Ultramafic Rocks of New Caledonia. *Environmental Science & Technology* **2009**, *43* (19), 7384-7390.
(6) Delina, R. E. G.; Perez, J. P. H.; Stammeier, J. A.; Bazarkina, E. F.; Benning, L. G. Partitioning and Mobility of Chromium in Iron-Rich Laterites from an Optimized Sequential Extraction Procedure. *Environmental Science & Technology* **2024**, *58* (14), 6391-6401.
(7) Webb, S. M. SIXpack: a graphical user interface for XAS analysis using IFEFFIT. *Physica Scripta* **2005**, *2005* (T115), 1011.
(8) Newville, M. IFEFFIT : interactive XAFS analysis and FEFF fitting. *Journal of Synchrotron Radiation* **2001**, *8* (2), 322-324.
(9) Kelly, S. D.; Hesterberg, D.; Ravel, B. Analysis of Soils and Minerals Using X-ray Absorption Spectroscopy. In *Methods of Soil Analysis Part 5—Mineralogical Methods*, A. L. Ulery, L. R. D. Ed.; Soil Science Society of America, Inc., 2008; pp 387-463.
(10) Downward, L.; Booth, C. H.; Lukens, W. W.; Bridges, F. A Variation of the F-Test for Determining Statistical Relevance of Particular Parameters in EXAFS Fits. *AIP Conference Proceedings* **2007**, *882*, 129-131.
(11) Gultom, T.; Sianipar, A. High pressure acid leaching: a newly introduced technology in Indonesia. *IOP Conference Series: Earth and Environmental Science* **2020**, *413*, 012015.
(12) Stanković, S.; Stopić, S.; Sokić, M.; Marković, B.; Friedrich, B. Review of the past, present, and future of the hydrometallurgical production of nickel and cobalt from lateritic ores. *Metallurgical and Materials Engineering* **2020**, *26* (2), 199-208.
(13) Shibayama, K.; Yokogawa, T.; Sato, H.; Enomoto, M.; Nakai, O.; Ito, T.; Mizuno, F.; Hattori, Y. Taganito HPAL Plant Project. *Minerals Engineering* **2016**, *88*, 61-65.
(14) Whittington, B. I.; Muir, D. Pressure Acid Leaching of Nickel Laterites: A Review. *Mineral Processing and Extractive Metallurgy Review* **2000**, *21* (6), 527-599.
(15) Ang, C. A.; Zhang, F.; Azimi, G. Waste Valorization Process: Sulfur Removal and Hematite Recovery from High Pressure Acid Leach Residue for Steelmaking. *ACS Sustainable Chemistry & Engineering* **2017**, *5* (9), 8416-8423.
(16) Whittington, B. I.; Johnson, J. A.; Quan, L. P.; McDonald, R. G.; Muir, D. M. Pressure acid leaching of arid-region nickel laterite ore: Part II. Effect of ore type. *Hydrometallurgy* **2003**, *70* (1-3), 47-62.
(17) Önal, M. A. R.; Topkaya, Y. A. Pressure acid leaching of Çaldağ lateritic nickel ore: An alternative to heap leaching. *Hydrometallurgy* **2014**, *142*, 98-107.
(18) Ucyildiz, A.; Girgin, I. High pressure sulphuric acid leaching of lateritic nickel ore. *Physicochemical Problems of Mineral Processing* **2017**, *53* (1), 475-488.
(19) Kaya, Ş.; Topkaya, Y. A. High pressure acid leaching of a refractory lateritic nickel ore. *Minerals Engineering* **2011**, *24* (11), 1188-1197.
(20) Dold, B. Speciation of the most soluble phases in a sequential extraction procedure adapted for geochemical studies of copper sulfide mine waste. *Journal of Geochemical Exploration* **2003**, *80* (1), 55-68.

(21) Claff, S. R.; Sullivan, L. A.; Burton, E. D.; Bush, R. T. A sequential extraction procedure for acid sulfate soils: Partitioning of iron. *Geoderma* **2010**, *155* (3-4), 224-230.
(22) Bokhari, S. N. H.; Meisel, T. C. Method Development and Optimisation of Sodium Peroxide Sintering for Geological Samples. *Geostandards and Geoanalytical Research* **2017**, *41* (2), 181-195.
